# Harnessing Transformation of Metal‐Ligand Coordination in Dinuclear Ni(II)‐Schiff Base Coordination Polymer for Promoting Electrochemical Oxygen Evolution

**DOI:** 10.1002/advs.202524014

**Published:** 2026-01-29

**Authors:** Ruei‐Hung Juang, Han‐Jung Li, Yu‐Chung Chang, Chieh‐Wei Chang, Ricky Yu‐Syun Fan, Kien Voon Kong, Jeng‐Lung Chen, Chia‐Shuo Hsu, Po‐Heng Lin, Chun‐Hong Kuo

**Affiliations:** ^1^ Department of Applied Chemistry National Yang Ming Chiao Tung University Hsinchu Taiwan; ^2^ Department of Chemistry Chung Yuan Christian University Taoyuan Taiwan; ^3^ Department of Materials Science and Engineering Feng Chia University Taichung Taiwan; ^4^ Department of Chemistry National Chung Hsing University Taichung Taiwan; ^5^ Department of Chemistry National Taiwan University Taipei Taiwan; ^6^ National Synchrotron Radiation Research Center Hsinchu Taiwan

**Keywords:** coordination, nickel, OER, schiff base, XAS

## Abstract

One of the major challenges in overall water splitting is improving the sluggish kinetics of the anodic oxygen evolution reaction (OER). Various 3d transition metal oxides, hydroxides, and complexes have been investigated as cost‐effective OER electrocatalysts. Herein, a novel type of Ni‐based coordination polymer, [Ni_2_(tmd)_2_]_n_, containing six‐coordinate Ni centers (two Ni─O and four Ni─N bonds) was synthesized. It acts as a pre‐catalyst for alkaline OER, undergoing structural transformation in KOH into [Ni_2_(tmd)_2_]_n_‐OH with five‐coordinate Ni centers, and further into the catalytically active state [Ni_2_(tmd)_2_]_n_‐CV after 150 cycles of cyclic voltammetry (CV). Under Fe‐depleted alkaline conditions (KOH, pH = 13.8), [Ni_2_(tmd)_2_]_n_‐CV exhibited outstanding OER performance, with overpotentials of η_10_ = 222 mV and η_50_ = 385 mV, a Tafel slope of 94.15 mV/dec, and a charge‐transfer resistance (R_ct_) of 3.1 Ω, lower than those of NiPc, RuO_2_, and NiO. The catalyst also manifested excellent durability, with only a 6.1% drop in current density after 100 h of operation. X‐ray absorption and emission spectroscopies revealed its structural changes at different applied potentials. These changes are attributed to adsorption of oxygen species at the five‐coordinate Ni sites, potentially associated with Ni–OOH intermediates forming during OER.

## Graphical abstract

Ni–Schiff base coordination polymer [Ni_2_(tmd)_2_]_n_ was synthesized and applied to enhance alkaline OER by virtue of its transformation between 5‐ and 6‐coordinate Ni sites along with the spin state exchange.

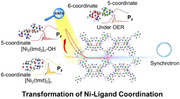

## 1 Introduction

In recent years, the electrochemical production of green hydrogen through water splitting, particularly via the hydrogen evolution reaction (HER), has emerged as a key technology for achieving net‐zero carbon emissions [1, 2]. However, the overall efficiency of water splitting is significantly hindered by the sluggish kinetics of the anodic oxygen evolution reaction (OER) [3]. As a result, substantial efforts have been dedicated to developing efficient electrocatalysts for OER [4]. Platinum‐group metals and their oxides (e.g., RuO_2_ and IrO_2_) exhibit excellent catalytic performance in OER. Nevertheless, their high cost and scarcity pose significant barriers to large‐scale industrial applications [5, 6]. In contrast, abundant 3d transition metal oxides and hydroxides have been explored as promising alternatives [7, 8, 9, 10]. However, these materials often suffer from low atomic utilization efficiency, poorly defined active sites, and uncontrollable surface transformations during catalysis [11, 12]. To address these limitations, growing attention has been directed toward designing 3d metal‐based electrocatalysts with well‐regulated metal–ligand coordination environments. Among the reported OER electrocatalysts, mononuclear metal coordination compounds (MMCs) have emerged, offering nearly 100% atomic utilization and precisely defined coordination environments [13]. In MMCs, the coordination geometry and metal–ligand bond strength are crucial factors that influence catalytic activity and stability. For instance, tuning the hybridization between ligand p‐orbitals and metal d‐orbitals can significantly modulate the catalytic performance [14, 15]. Compared to MMCs, dinuclear metal coordination compounds (DMCs) have recently been reported to exhibit higher spatial densities of active sites and potentially superior OER performance compared to MMCs [16]. Despite synthetic challenges and low yields, rationally designed DMCs with tailored metal–ligand electronic effects offer a promising approach to overcoming current catalyst limitations and providing new insights into the design of OER.

Nickel (Ni), an abundant, low‐cost, and environmentally friendly 3d transition metal, has been extensively investigated for electrocatalytic water splitting [17]. Ni‐based materials demonstrate promising catalytic activity for the oxygen evolution reaction (OER), primarily due to nickel's moderate oxygen affinity and tunable oxidation state, enabling effective participation in the four‐electron transfer pathway of OER [18]. However, many Ni‐based dinuclear metal complexes (DMCs) function as unstable pre‐catalysts, often transforming into nickel oxides or hydroxides under OER conditions [19]. For instance, Azadi et al. reported a tetranuclear Ni(II) complex that oxidized into NiO during OER [20]. Similarly, Anamika et al. synthesized heteroleptic Ni(II) complexes as OER electrocatalysts, which ultimately converted into nickel oxide (NiO_x_) and nickel hydroxide (Ni(OH)_2_) after the reaction [21]. In another study, Lichen Bai et al. developed a planar, tetracoordinated Ni–N–C single‐atom catalyst. In situ X‐ray absorption spectroscopy (XAS) revealed that nitrogen ligands were partially replaced by oxygen atoms after immersion in KOH. During the OER process, trace Fe impurities in commercial KOH were found to bridge with Ni through oxygen atoms, forming a Ni–Fe diatomic catalyst that significantly enhanced OER performance [22]. These findings highlight that the highly oxidative environment of OER can induce substantial structural changes in the original catalyst materials. Therefore, a major goal in the catalyst design is to rationally engineer pre‐catalyst structures that controllably transform into highly active and stable species upon electrochemical activation. Achieving this would represent a significant advancement toward durable and efficient OER electrocatalysts.

Coordination polymers (CPs) have attracted intense attention due to their tunable structures and wide range of multifunctional applications, including selective gas adsorption, catalysis, and oxidation reactions [23, 24, 25, 26]. Owing to the high natural abundance and low cost of nickel, nickel‐based CPs have demonstrated considerable catalytic potential in various organic transformations [27, 28]. Among the ligands used in constructing these materials, Schiff base ligands are particularly prominent. They provide versatile coordination environments that critically influence both the structural characteristics and catalytic performance of Ni‐based CPs [29, 30]. Schiff base ligands also offer synthetic advantages due to the high reactivity of their aldehyde and amine precursors, allowing for the rational design of ligands as secondary building units (SBUs) or for post‐synthetic modification of CP networks [31, 32, 33, 34]. Notably, several Ni‐based CPs incorporating Schiff base ligands have been synthesized and shown promising catalytic activity in oxygen evolution reactions [35, 36, 37]. However, most of these CPs still rely heavily on conventional carboxylate groups to link the inorganic nodes, which restricts the structural diversity and limits their broader functional applications.

In this work, we report the synthesis of a novel Schiff base ligand, H_2_tmd (see Experimental Section and Scheme S1), featuring an O, N, N, O coordination pocket that enables distinct coordination to nickel ions. This ligand facilitates the formation of a new type of Ni‐based coordination polymer (CP) composed by a Ni–Schiff base network [38, 39, 40, 41]. The resulting Ni–Schiff base CP, [Ni_2_(tmd)_2_]_n_, exhibited an octahedral six‐coordinate geometry at each Ni center, consisting of two Ni─O and four Ni─N bonds. This compound served as a pre‐catalyst for alkaline electrochemical oxygen evolution reaction (OER). Upon immersion in KOH, it underwent transformation into [Ni_2_(tmd)_2_]_n_‐OH, in which the Ni centers adopted a square pyramid five‐coordinate geometry. Further electrochemical activation through 150 cycles of cyclic voltammetry (CV) resulted in the formation of the catalytically active phase, [Ni_2_(tmd)_2_]_n_‐CV. Under Fe‐depleted alkaline conditions (KOH, pH 13.8), the activated [Ni_2_(tmd)_2_]_n_‐CV catalyst exhibited excellent OER performance with significantly lower overpotentials (η_10_ = 222 mV and η_50_ = 385 mV), a Tafel slope of 94.15 mV/dec, and a charge‐transfer resistance (R_ct_) of 3.1 Ω, surpassing the performances of benchmark catalysts such as NiPc, RuO_2_, and NiO. The catalyst also manifested outstanding durability, maintaining performance over 100 h with only 6.1% decrease in current density. In situ XAS analysis showed the gradual changes in the Ni K‐edge features and an increase in the Ni–O_ads_ coordination number, indicating the formation of oxygenated intermediates and local structural rearrangement during OER. Complementary XES results revealed a reversible spin‐state change of Ni^2+^ in [Ni_2_(tmd)_2_]_n_‐CV from high‐spin to low‐spin from, driven by oxygen adsorption that restores the octahedral geometry. Supported by simulations, these findings clarified the structural evolution of [Ni_2_(tmd)_2_]_n_ and confirmed that the OER follows the adsorbate evolution mechanism (AEM), where oxygen intermediates preferentially bind to one of the Ni centers in the binuclear unit.

## 2 Results and Discussion

### 2.1 Structural Analyses of [Ni_2_(tmd)_2_]_n_

**FIGURE 1:**
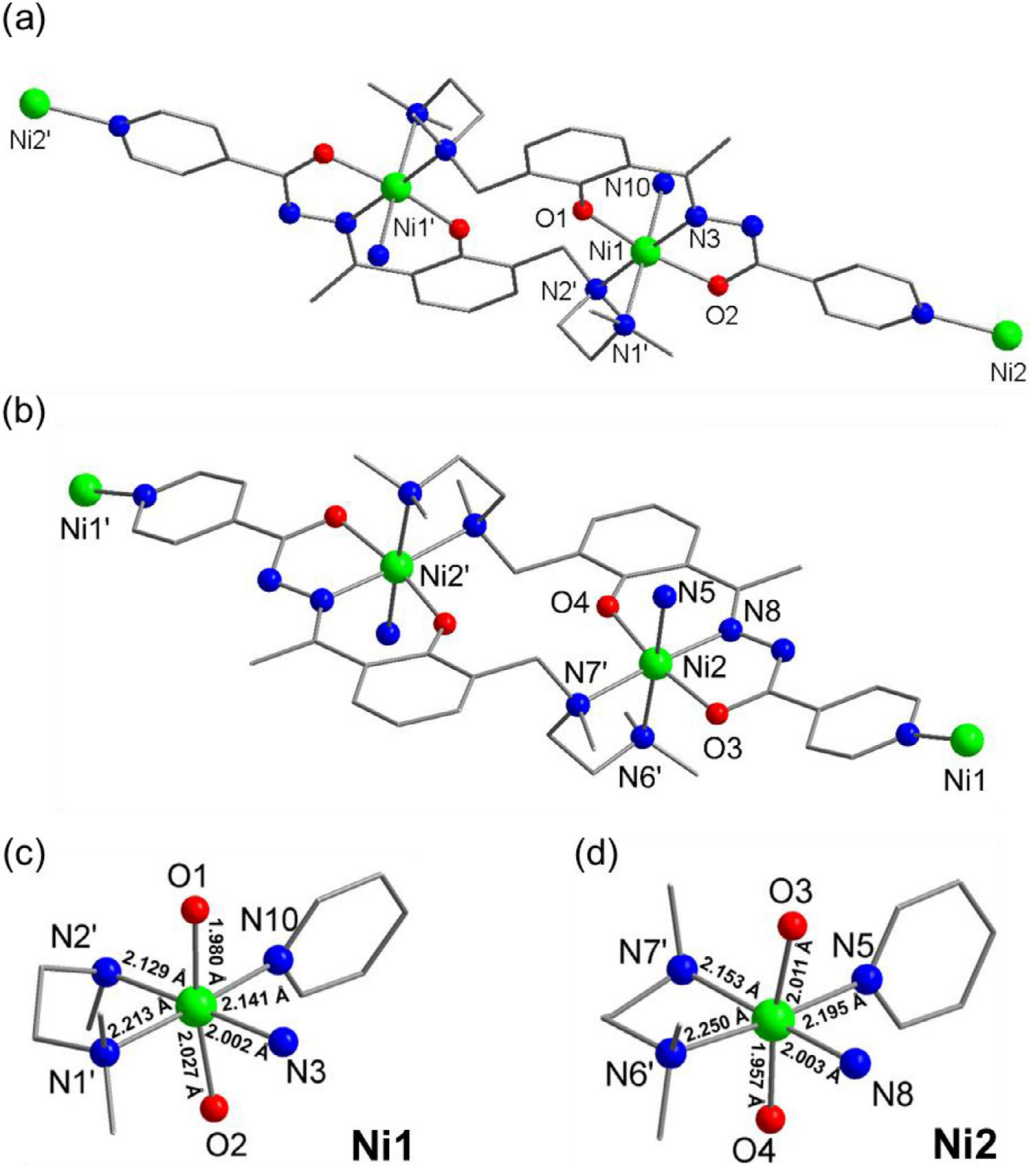
(a, b) The molecular structure of [Ni_2_(tmd)_2_]_n_ with the drawn mirror‐symmetry centers of (a) Ni1 and Ni1', and (b) Ni2 and Ni2'. (c, d) Metal‐oxido/nitrogen distances of (c) Ni1 and (d) Ni2. Color code: green (Ni), red (O), blue (N), and gray (C). H atoms and solvent molecules are omitted for clarity.

Polydentate Schiff base ligands derived from *o*‐vanillin and hydrazine have been widely utilized in the synthesis of metal complexes. These ligands have enabled the formation of a broad range of structures, from mononuclear to multinuclear complexes, incorporating 3d transition metals (e.g., Cu^2^^+^, Mn^2^^+^/Mn^3^^+^) and 4f lanthanides (e.g., Dy^3^^+^). The possible coordination modes are illustrated in Scheme S2. Pyridine, acting as a linker, further facilitates the extension of inorganic units into polymeric network structures. In this study, we employed an O, N, O‐based multidentate chelating system and introduced ethylenediamine as an additional bidentate coordination site to construct a novel Ni‐based coordination polymer. Single‐crystal X‐ray diffraction (SCXRD) analysis revealed that the resulting complex crystallizes in the orthorhombic space group *Ibca*. The structure features mirror‐symmetric dinuclear Ni cores (Ni1/Ni1' and Ni2/Ni2'), where each pair of Ni^2+^ ions is coordinated within the binding pockets of two tmd^2‒^ ligands, forming the [Ni_2_(tmd)_2_] unit (Figure 1a,b). Both Ni^2^^+^ centers (Ni1 and Ni2) adopt octahedral coordination geometries, each coordinated by two oxygen atoms and one nitrogen atom from the tmd^2^^−^ ligand (O1, O2, and N3 for Ni1; O3, O4, and N8 for Ni2), with highly comparable local coordination environments (Figure 1c,d). The remaining three coordination sites are occupied by two amine nitrogen atoms from a neighboring ligand (N1′ and N2′ for Ni1; N6′ and N7′ for Ni2) and one from another neighboring ligand (N10 for Ni1 and N5 for Ni2). Notably, the Ni···Ni separations between two neighboring Ni1 and Ni2 centers are 6.299 and 6.261 Å, respectively, which are longer than those observed in typical dinuclear Ni complexes. Selected bond lengths and angles are listed in Table S1, confirming the structural similarity between the two Ni^2^^+^ centers. Each Ni^2^^+^ ion is connected to three neighboring Ni^2^^+^ ions, generating an extended 3D coordination network (Figure S1). Two distinct types of channels are observed along the *b*‐axis, with edge spans of 14.709, 15.328, and 8.810 Å.

**FIGURE 2:**
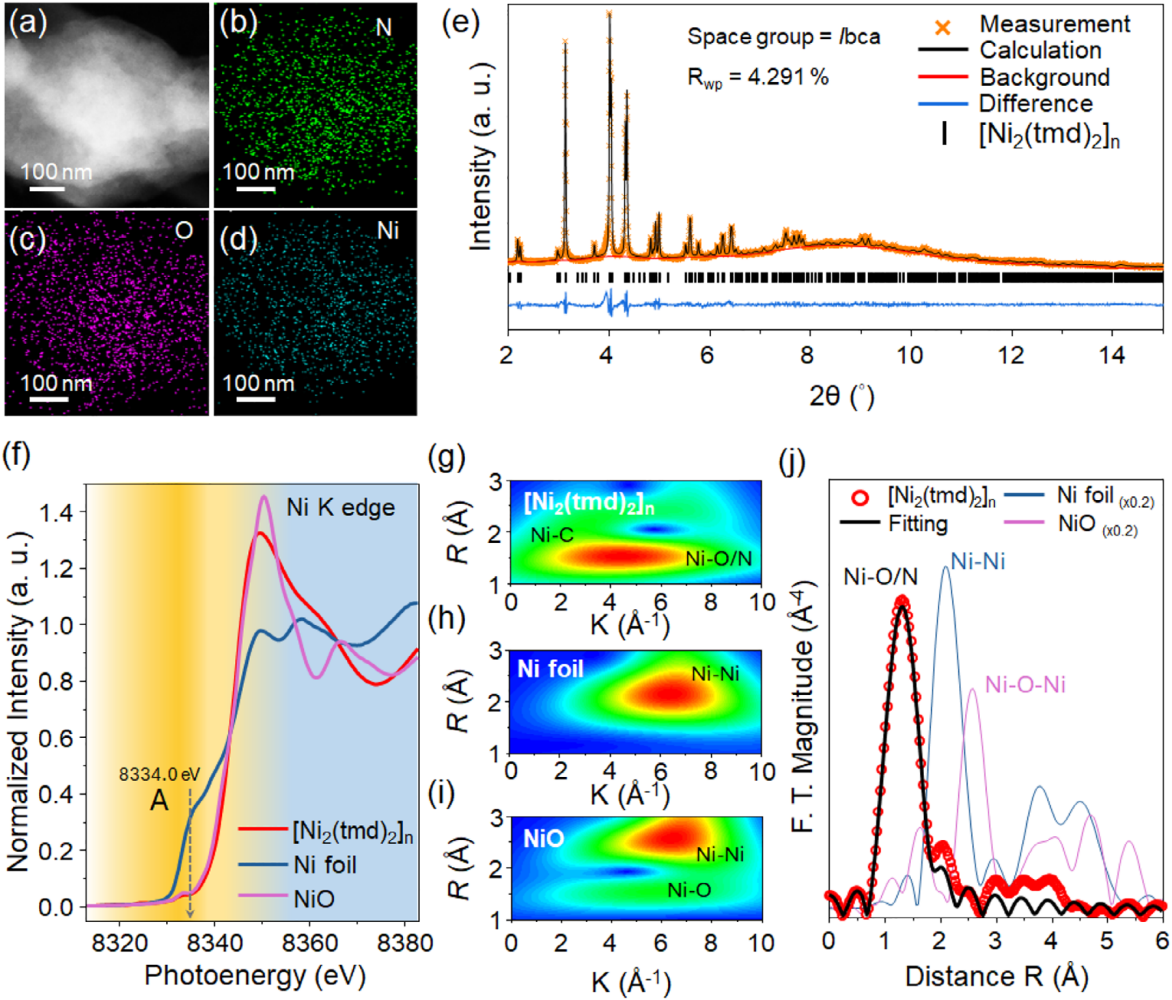
(a) HAADF‐STEM and (b–d) EDS elemental maps (N, O, Ni) for [Ni_2_(tmd)_2_]_n_. (e) High‐resolution PXRD pattern with refinement results for [Ni_2_(tmd)_2_]_n_. (f) Ni K‐edge XANES spectra of [Ni_2_(tmd)_2_]_n_, Ni foil, and NiO. (g–i) Wavelet transforms of (g) [Ni_2_(tmd)_2_]_n_, (h) Ni foil, and (i) NiO. (j) Ni K‐edge EXAFS fitting results for [Ni_2_(tmd)_2_]_n_.

Figure 2a–d shows the high‐angle annular dark‐field scanning transmission electron microscopy (HAADF‐STEM) image of [Ni_2_(tmd)_2_]_n_ powder, along with the corresponding STEM–EDS elemental maps for (b) nitrogen, (c) oxygen, and (d) nickel. These images confirm the homogeneous distribution of N, O, and Ni elements throughout the sample and demonstrate the structural stability of [Ni_2_(tmd)_2_]_n_ under electron beam irradiation. Inductively coupled plasma mass spectrometry (ICP–MS) analysis revealed that Ni atoms account for 0.38% of the total composition. To further elucidate the average crystal structure of [Ni_2_(tmd)_2_]_n_, high‐resolution powder X‐ray diffraction (HRPXRD) measurements were performed using synchrotron radiation with an energy of 20 keV (λ = 0.61992 Å), followed by Rietveld refinement, as shown in Figure 2e. The refinement yielded a low weighted profile R‐factor (R_wp_) of less than 5%, indicating that the powder sample retains a well‐ordered crystalline structure consistent with the single‐crystal XRD data. Detailed Rietveld refinement parameters are provided in Table S2.

In addition to crystal structure analysis, X‐ray absorption spectroscopy (XAS) was employed to gain insight into the electronic structure and local coordination environment of [Ni_2_(tmd)_2_]_n_. XAS probes electronic transitions from core levels to unoccupied states above the Fermi level, providing valuable information on oxidation states and coordination geometries. Figure 2f displays the X‐ray absorption near‐edge structure (XANES) spectra of [Ni_2_(tmd)_2_]_n_, Ni foil, and NiO. A pre‐edge feature attributed to the 1s → 3d electronic transition (Peak A, pink dashed line) appears around 8334.0 eV. The relatively low intensity of this pre‐edge feature in [Ni_2_(tmd)_2_]_n_ suggests a highly symmetric local coordination environment around the Ni centers. The first derivative XANES spectra provide insight into the oxidation state of Ni in the samples. In general, the absorption edge shifts to higher energies with increasing Ni valence. As shown in Figure S2, the derivative peak of NiO appears at 8345 eV. The corresponding peak for [Ni_2_(tmd)_2_]_n_ is located at 8344.1 eV, only slightly lower in energy. This negligible difference suggests that the Ni centers in [Ni_2_(tmd)_2_]_n_ adopt an oxidation state very close to Ni^2^^+^. This is consistent with the local coordination number of six, as confirmed by extended X‐ray absorption fine structure (EXAFS) fitting (Table S3).

We conducted a comparative analysis of the wavelet transforms of the Fourier‐transformed extended X‐ray absorption fine structure (EXAFS) spectra to gain further insight into the local coordination environments. This technique enables rapid comparison of the 2D distributions in momentum space (k‐space) and real space (R‐space) across different materials, providing a clearer view of the contributions from various scattering paths to the overall EXAFS signal. Figure 2g–i displays the wavelet transform maps for [Ni_2_(tmd)_2_]_n_, Ni foil, and NiO, respectively. For Ni foil (Figure 2h), the dominant scattering path arises from Ni─Ni bonds, located at (k, R) = (6.5 Å^−1^, 2.17 Å), indicating an average Ni─Ni bond length of 2.17 Å. In the case of NiO (Figure 2i), two primary scattering paths are observed: the first coordination shell Ni–O at (k, R) = (5.9 Å^−1^, 1.53 Å) and the second coordination shell Ni–Ni at (k, R) = (6.55 Å^−1^, 2.6 Å). These features reflect a local environment dominated by oxygen atoms, with an average Ni─O bond length of 1.53 Å. In contrast, the wavelet transform of [Ni_2_(tmd)_2_]_n_ (Figure 2g) reveals a main scattering path at (k, R) = (4.6 Å^−^^1^, 1.52 Å) corresponding to Ni─N/O bond. At (k, R) = (3.1 Å^−^^1^, 2.21 Å) corresponding to multiple scattering Ni─C bond. To further elucidate the coordination environment, we performed EXAFS data and fitted the first and second coordination shells (1.1 Å < R < 2.8 Å). The k^3^‐weighted Fourier‐transformed EXAFS spectrum and corresponding fit are shown in Figure 2j, with the fit indicated by the black line. The extracted structural parameters are summarized in Table S3. These results confirm that the local coordination structure of [Ni_2_(tmd)_2_]_n_ in powder form features an octahedral six‐coordinate geometry, consisting of two Ni–O and four Ni─N bonds, in excellent agreement with the single‐crystal structure.

### 2.2 Structural Change of [Ni_2_(tmd)_2_]_n_ Immersed in the Alkaline Electrolyte

**FIGURE 3:**
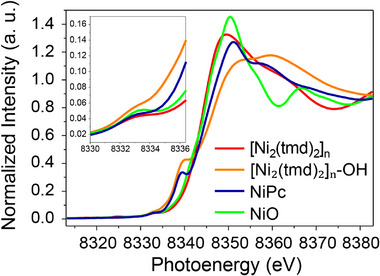
Ni K‐edge XANE spectra of [Ni_2_(tmd)_2_]_n_, [Ni_2_(tmd)_2_]_n_‐OH, NiPc, and NiO.

In this study, the as‐synthesized [Ni_2_(tmd)_2_]_n_ was employed as the pre‐catalyst for all alkaline oxygen evolution reaction (OER) experiments. To gain preliminary insight into the structural evolution of [Ni_2_(tmd)_2_]_n_ in an alkaline electrolyte (1 m KOH with depleted Fe ions), we immersed the [Ni_2_(tmd)_2_]_n_ powder in 1 m KOH for 1 h, followed by X‐ray absorption spectroscopy (XAS) measurements. As shown in the X‐ray absorption near‐edge structure (XANES) spectra (Figure S3), the pre‐edge intensity at 8334.0 eV increases after KOH treatment (the inset), indicating a reduction in coordination symmetry. A characteristic peak emerges at 8340.0 eV, corresponding to the 1s → 4p_z_ electronic transition. These findings imply that under strong alkaline conditions, the pre‐catalyst does not preserve its original octahedral six‐coordinate geometry, but instead evolves into an alternative coordination environment, likely involving a reduction in coordination number [42]. This transformed complex is denoted as [Ni_2_(tmd)_2_]_n_‐OH. Figure 3 compares the XANES spectra of [Ni_2_(tmd)_2_]_n_ and [Ni_2_(tmd)_2_]_n_‐OH (orange line) with those of a known planar Ni–N four‐coordinate compound, nickel phthalocyanine (NiPc, blue line), and commercial NiO. The 1s → 4p_z_ transition intensity in [Ni_2_(tmd)_2_]_n_‐OH is higher than that in NiPc, which may be attributed to the presence of both N and O atoms in the first coordination shell, or to subtle deviations of its coordination environment from an ideal square‐planar geometry. To further clarify this phenomenon, additional characterization techniques were employed in the following studies.

### 2.3 XAS Simulation

**FIGURE 4:**
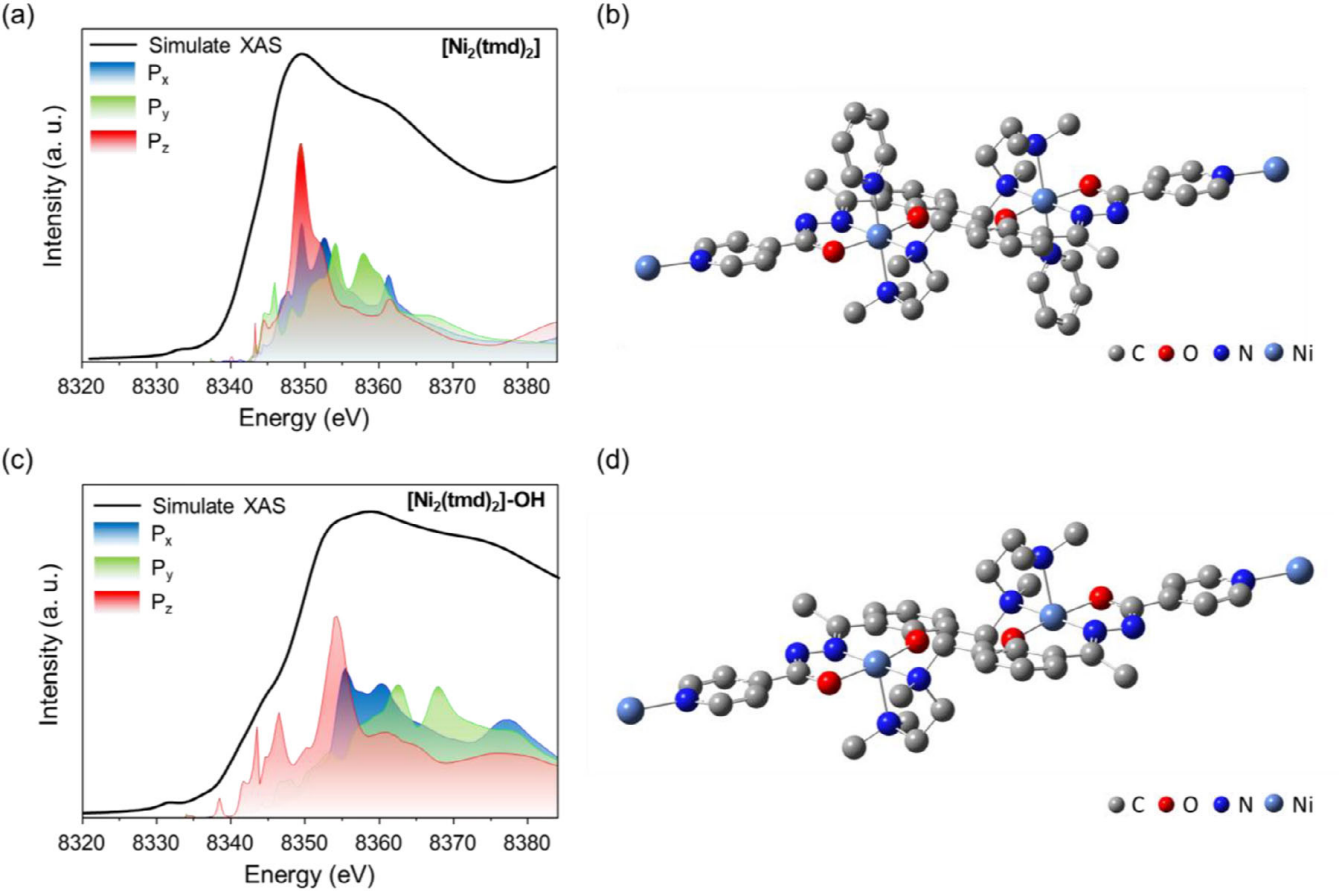
FDMNES simulations and structures of (a, b) [Ni_2_(tmd)_2_]_n_ and (c, d) [Ni_2_(tmd)_2_]_n_‐OH.

We aimed to validate the variations observed in the XANES features under aqueous conditions (Figure 3), and thus performed FDMNES simulations on [Ni_2_(tmd)_2_]_n_ (dry powder). To examine whether the coordination number of [Ni_2_(tmd)_2_]_n_ decreased in KOH solution, we systematically reduced the number of ligands in the structural models and compared the simulated spectra with the experimental data. Furthermore, by analyzing the projected density of states (p‐DOS) in conjunction with the simulated spectra, we were able to assign the individual spectral features to their corresponding electronic states. In Figure 4a, the projected DOS of the Ni 4p orbitals (P_x_, P_y_, and P_z_) shows that these components largely overlap, with the dominant contribution arising from the P_z_ orbital, which exhibits a pronounced peak around 8350 eV. This feature originates from the nearly ideal octahedral coordination environment in the pristine structure (Figure 4b). Upon dissolution in KOH solution, however, a new feature emerges at approximately 8340 eV, while the intensity at 8350 eV diminishes due to the broader dispersion of the P_z_ contribution (Figure 4c). Specifically, the coordination number decreases to five, leading to a redistribution of the Ni 4p orbitals, particularly the P_z_ states (Figure 4d). Importantly, these simulated results are in good agreement with the experimental XANES spectra, and the decrease in coordination number is consistent with the structural changes independently revealed by the EXAFS analysis. This spectral evolution reflects the reduced coordination environment and the resulting anisotropy of the Ni 4p orbitals. Collectively, the results indicate a clear symmetry lowering, with the local coordination geometry transformed from an octahedral environment to a square‐pyramidal configuration. Such a reduction in coordination number is consistent with the expected ligand lability of Ni in alkaline media and provides direct spectroscopic evidence for structural transformation under electrochemically relevant conditions.

To further verify the consistency between the XANES simulation and the actual local coordination environment, Figure S4 presents the χ(R) k^3^‐weighted FT‐EXAFS spectra derived from Figure 3. The Ni─N/O bond length of [Ni_2_(tmd)_2_]_n_‐OH (*n* = 5) lies between that of [Ni_2_(tmd)_2_]_n_ (N = 6) and NiPc (N = 4), indicating that a decrease in coordination number results in a slight contraction of the metal–ligand bond length. Moreover, no feature corresponding to the second‐shell Ni–O–Ni scattering of NiO were observed, suggesting that no Ni(OH)_2_ or NiO byproducts were formed during the structural transformation. To obtain more accurate structural parameters, the first coordination shell of the model was incorporated into the EXAFS fitting. Considering that excessive fitting paths may reduce the degrees of freedom, the Ni–N coordination paths were separated into long (Ni_1_–N_1_/N_10_) and short (Ni_1_–N_2_/N_3_) distances, denoted as Ni–N(L) and Ni–N(S), respectively. Table S3 and Figure S5 collect the fitting results of [Ni_2_(tmd)_2_]_n_‐OH, NiPc, NiO, and Ni foil. Figure S5a present the fitting results of [Ni_2_(tmd)_2_]_n_‐OH, where the Ni–O, Ni–N(S), and Ni─N(L) bond lengths are 1.744 Å (*n* = 2.004), 1.946 Å (*n* = 2.053), and 2.302 Å (*n* = 1.090), respectively. These values are in excellent agreement with the simulated model, confirming that in an alkaline environment, the pyridine nitrogen atoms preferentially dissociate, leading to the formation of a tetrahedral coordination geometry. Notably, the equal lengths of the four Ni─N bonds in NiPc (1.910 Å) infer its lower coordinate number than that of [Ni_2_(tmd)_2_]_n_‐OH (Figure S5b). The structure of [Ni_2_(tmd)_2_]_n_‐OH also differs from the NiO with Ni─O and Ni─Ni bonds (Figure S5c) and metallic Ni foil with Ni─Ni bonds (Figure S5d).

### 2.4 Electrochemical Activation of [Ni_2_(tmd)_2_]_n_

Prior to the OER measurements, the working electrode loaded with [Ni_2_(tmd)_2_]_n_ was fabricated and subjected to electrochemical activation through cyclic voltammetry (CV), as described in the Experimental Section. This CV activation process was used to monitor surface changes of the electrode during repeated redox cycling. Notably, before initiating the CV test, the [Ni_2_(tmd)_2_]_n_ electrode was immersed in 1 m KOH to induce the transformation into [Ni_2_(tmd)_2_]_n_‐OH. The CV measurements were conducted within the typical OER potential window of 0.9 to 2.0 V vs. RHE. As shown in Figure S6a, the current density increased progressively with the number of CV cycles and reached a steady state after approximately 100 cycles. According to the Randles–Ševčík equation, the peak current of a redox process is directly proportional to the concentration of electroactive species on the electrode under consistent scan rate and reaction conditions [43]. The inset in Figure S6a provides an enlarged view of the redox peaks in the potential range of 1.10–1.45 V vs. RHE, where the redox peak currents are observed to increase with successive cycles. This trend indicates a growing number of electrochemically active sites on the electrode surface. To further evaluate the electrocatalytic performance, Tafel analysis was conducted using the anodic currents obtained from CV at various cycle numbers. As shown in Figure S6b, the Tafel slope markedly decreases with increasing cycle number, which is consistent with the rise in CV current. This clearly reflects a substantial enhancement in the OER kinetics. All corresponding overpotential and Tafel slope values are summarized in Table S4.

The observed changes in the CV measurements prompted us to further investigate the behavior of [Ni_2_(tmd)_2_]_n_‐OH during the electrochemical activation process. To this end, electrochemical impedance spectroscopy (EIS) was performed, and the resulting Bode plots were analyzed to examine the frequency response characteristics of the electrode surface. In Bode plots, the high‐frequency region corresponds to the Faradaic process (i.e., redox activity of the catalyst), while the low‐frequency region is associated with the non‐Faradaic process (specifically, double‐layer charging and ion diffusion) [44]. Figure S7a displays the Bode plots of [Ni_2_(tmd)_2_]_n_‐OH at open‐circuit potential (OCP) across different CV cycle numbers. In the high‐frequency region (e.g., at log(f) = 4, where *f* denotes the frequency of the applied AC potential), the phase angle (θ) becomes less negative with increasing cycle number, indicating a pronounced change in the Faradaic behavior of the catalyst. Figure S7b provides a schematic illustration of this phenomenon, where E(t) represents the time‐dependent applied potential, I(t) the resulting current response, t time, ω the angular frequency (2πf), and φ the phase difference. As shown, the large initial phase difference (φ) between E(t) and I(t) at the first CV cycle reflects high impedance in the early stage of [Ni_2_(tmd)_2_]_n_‐OH. After 150 CV cycles, the phase difference at log(f) = 4 approaches zero, signifying increased synchrony between E(t) and I(t), a clear indication of reduced impedance and improved electron transfer kinetics. Figure S7c shows the variation of double‐layer capacitance C′(ω) with log(f). In the low‐frequency region (e.g., log(f) = −2), C′(ω) increases significantly from 0.2 to 4.08 mF as the number of CV cycles increases. This enhancement is attributed to improved mass transport kinetics in the non‐Faradaic process. Figure S7d conceptually illustrates this improvement, showing that the diffusion of the ionic double layer at the electrode–electrolyte interface becomes progressively more efficient from the 1st to the 150th CV cycle.

In addition to probing reaction kinetics via electrochemical impedance spectroscopy, in situ XAS measurements were conducted to investigate the local structural evolution of [Ni_2_(tmd)_2_]_n_‐OH during the CV activation process. For consistency, all XAS spectra were normalized by setting the absorbance to 1.0, as shown in Figure S8 [45]. The in situ experimental parameters mirrored those of the standard CV experiments. At selected cycle numbers (e.g., the 10th cycle), the CV scan was paused, and XAS data were collected under the corresponding applied potential. Figure S9a displays the Ni K‐edge XANES spectra of [Ni_2_(tmd)_2_]_n_, [Ni_2_(tmd)_2_]_n_‐OH, and samples subjected to various CV cycle counts. A notable spectral change occurs upon immersion in 1 M KOH, marking the transition from [Ni_2_(tmd)_2_]_n_ to [Ni_2_(tmd)_2_]_n_‐OH. After this transformation, the spectra remain largely unchanged, even after multiple CV cycles. To further analyze this, we enlarged region A and examined the photoenergy at which the normalized intensity reaches 0.5 (Figure S9b). A shift of 0.4 eV toward higher energy is observed upon conversion to [Ni_2_(tmd)_2_]_n_‐OH, accompanied by the appearance of a pre‐edge feature at ∼8339 eV. These spectral differences reflect a change in the local coordination environment of Ni. On the other hand, the lack of significant spectral variation after CV activation confirms the structural stability of the five‐coordinate [Ni_2_(tmd)_2_]_n_‐OH complex. Figure S10 presents wavelet transform (WT) analyses comparing [Ni_2_(tmd)_2_]_n_, [Ni_2_(tmd)_2_]_n_‐OH, the 150‐cycle CV‐treated [Ni_2_(tmd)_2_]_n_‐OH, and nickel phthalocyanine (NiPc). The primary scattering path for [Ni_2_(tmd)_2_]_n_ is centered at (k, R) = (4.6 Å^−^^1^, 1.52 Å), while that for [Ni_2_(tmd)_2_]_n_‐OH shifts to (4.9 Å^−^^1^, 1.52 Å), indicating a change in coordination. By comparison, NiPc shows a main scattering path at (k, R) = (5.5 Å^−^^1^, 1.3 Å), suggesting that [Ni_2_(tmd)_2_]_n_‐OH has longer Ni─N/O bond lengths than NiPc. After 150 CV cycles, the scattering path appears at (k, R) = (5.1 Å^−^^1^, 1.31 Å), closely matching that of [Ni_2_(tmd)_2_]_n_‐OH, indicating that the coordination structure remains largely preserved. Further insight was gained by fitting the EXAFS spectra of [Ni_2_(tmd)_2_]_n_‐OH collected at different CV cycle numbers (Figure S11), with fitting results summarized in Table S5. The Ni–O, Ni–N(S) and Ni‐N(L) coordination numbers remained unchanged after 150 CV cycles, indicating that [Ni_2_(tmd)_2_]_n_ transformed into [Ni_2_(tmd)_2_]_n_‐OH, exhibited good reversibility within the OER potential window. This is further supported by the absence of a significant shift in the redox peaks during the CV cycling shown in Figure S9a, suggesting that the active sites of [Ni_2_(tmd)_2_]_n_‐OH do not undergo irreversible reactions.

The local structural transformation of [Ni_2_(tmd)_2_]_n_, primarily involving Ni–N coordination changes, was analyzed in depth via XAS. Meanwhile, the XPS N 1s spectra were also recorded as supporting evidence. Figure S12a shows the potential coordination modes of [Ni_2_(tmd)_2_]_n_ with three kinds of N marked in different colors. Figure S12b displays the corresponding N 1s spectra of [Ni_2_(tmd)_2_]_n_ before and after CV activation for the colored N, which can be deconvoluted into four peaks corresponding to sp^2^ N of pyridine and imine (Ni‐N_Py/Im_, blue), N–N linkages (N‐N, red), tertiary amines (Ni–N_amine_, green), and nitrate functional groups originating from coordinatively unsaturated surface sites (NO_x_, pink). Following KOH treatment and CV activation, the sp^2^ N peak of [Ni_2_(tmd)_2_]_n_ exhibits a shift toward lower binding energy by 0.48 eV. Combined with XAS data, we propose that this results from the cleavage of the Ni─N bond and the subsequent formation of ionic bonds between pyridine and K^+^ ions in the electrolyte. This is attributed to the high polarizing power of Ni, which initially reduces the electron density of the N atom. Once the Ni─N bond disappears and pyridine forms ionic bonds with the abundant K^+^ in the electrolyte (Figure S12a), the electron density of the N atom increases, leading to the observed shift toward lower binding energy [46]. Furthermore, we utilized the relative peak areas from deconvolution to evaluate the changes in N‐containing functional groups before and after the structural transformation. The corresponding peak areas and relative ratios are summarized in Table S6. By comparing with the N‐group area ratios of [Ni_2_(tmd)_2_]_n_ and [Ni_2_(tmd)_2_]_n_‐CV, the values of N–N/Ni–N_Py/Im_ and Ni–N_amine_/Ni–N_Py/Im_ decrease after CV activation, while that of NO_x_/Ni–N_Py/Im_ elevates. It is very likely attributed to the Ni–OOH generated on the surface during OER, which facilitates the irreversible transformation of certain N‐containing functional groups into nitrate species. This may be attributed to the Ni–OOH generated on the surface during OER, which facilitates the irreversible transformation of certain N‐containing functional groups into nitrate species.

### 2.5 Electrocatalytic Alkaline Oxygen Evolution Reaction

**FIGURE 5:**
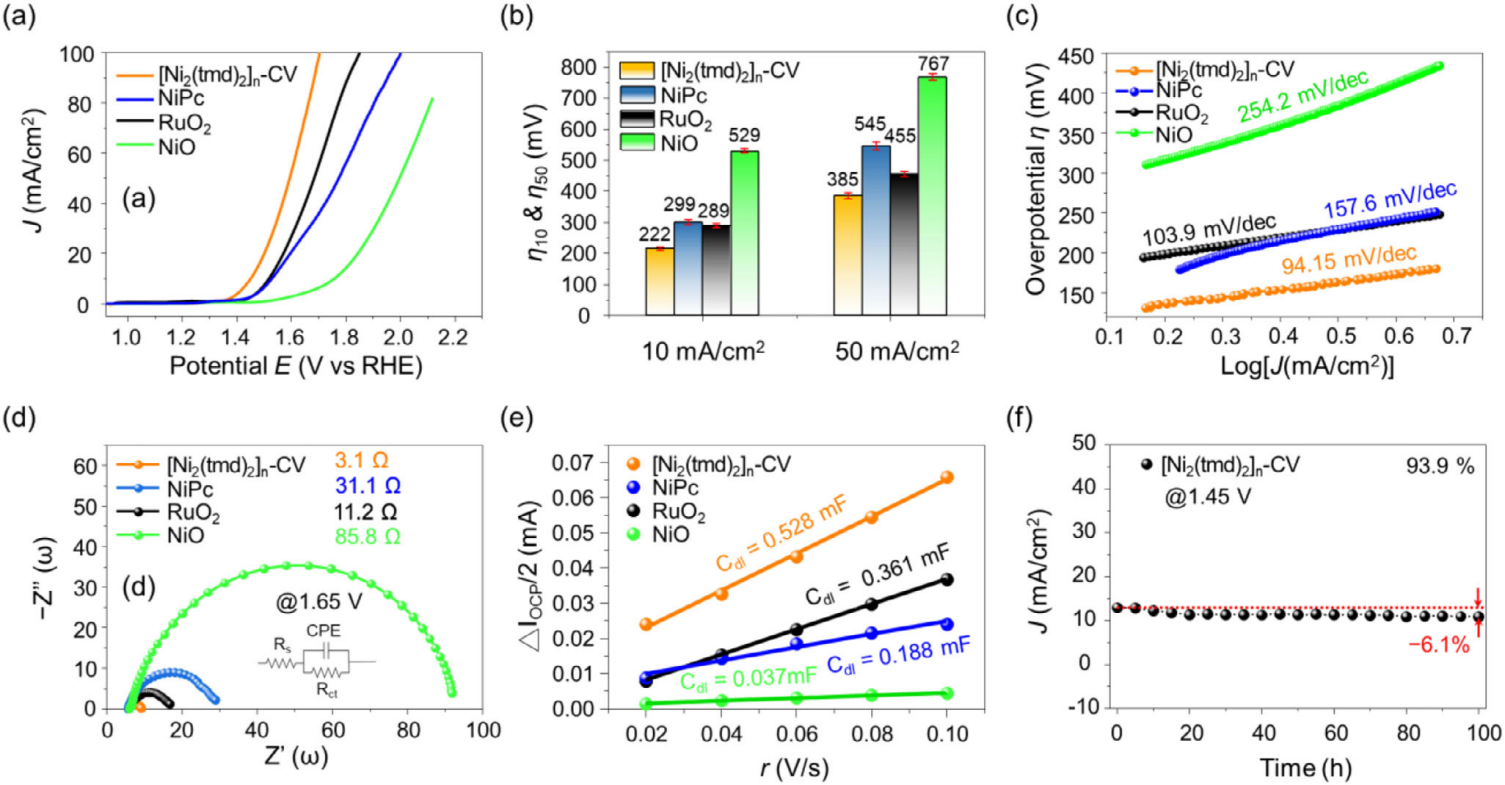
(a) LSV curves, (b) values of overpotentials at 10 and 50 mA/cm^2^, (c) Tafel plots, (d) Nyquist plots at 1.65 V, and (e) C_dl_ values for [Ni_2_(tmd)_2_]_n_‐CV, NiPc, RuO_2_, and NiO. (f) 100 h durability test at 1.45 V for [Ni_2_(tmd)_2_]_n_‐CV.

Since [Ni_2_(tmd)_2_]_n_‐OH can be activated through cyclic voltammetry (CV), the sample after 150 CV cycles (denoted as [Ni_2_(tmd)_2_]_n_‐CV) was selected as the electrocatalyst for alkaline OER measurements. Its performance was compared with those of NiPc, commercial RuO_2_, and commercial NiO. Figure 5a presents the linear sweep voltammetry (LSV) curves of [Ni_2_(tmd)_2_]_n_‐CV, NiPc, RuO_2_, and NiO, while Figure 5b summarizes their overpotentials (η) at the current densities (J) of 10 and 50 mA/cm^2^. At 10 mA/cm^2^, the overpotentials (η_10_) for [Ni_2_(tmd)_2_]_n_‐CV, NiPc, RuO_2_, and NiO are 220, 299, 289, and 529 mV, respectively. At 50 mA/cm^2^, the corresponding overpotentials (η50) are 360, 545, 455, and 767 mV. Among the four electrocatalysts, [Ni_2_(tmd)_2_]_n_‐CV shows the lowest η_10_ and η_50_ values, indicating it requires the lowest energy input to drive the OER under alkaline conditions. Figure 5c plots η versus log(J) to reveal the Tafel slopes, which are 94.15 mV/dec for [Ni_2_(tmd)_2_]_n_‐CV, 105.3 mV/dec for RuO_2_, 129.7 mV/dec for NiPc, and 158.2 mV/dec for NiO. The Tafel slope is a key indicator of reaction kinetics in electrocatalysis; thus, the smallest slope for [Ni_2_(tmd)_2_]_n_‐CV signifies the most favorable OER kinetics among the materials tested.

To further assess the electrocatalytic performance, electrochemical impedance spectroscopy (EIS) was performed. Figure 5d and Figure S13 display the Nyquist plots recorded at 1.65 V vs. RHE for [Ni_2_(tmd)_2_]_n_‐CV, NiPc, RuO_2_, and NiO. The semicircles observed in the high‐frequency region correspond to the charge‐transfer resistance at the electrode–electrolyte interface. The equivalent circuit model used for data fitting is illustrated in the inset of Figure 5d, with the corresponding fitting parameters summarized in Table S7. The circuit model comprises the solution resistance (R_s_), the charge‐transfer resistance (R_ct_), and a constant phase element (CPE). The CPE represents non‐ideal capacitive behavior and includes two components: CPE_T1_, which accounts for pseudo‐capacitance, and CPE_P1_, an exponent ranging between 0 and 1 that describes the deviation from ideal capacitive behavior. As shown in Table S7, the R_ct_ values follow the order: [Ni_2_(tmd)_2_]_n_‐CV (3.1 Ω) < RuO_2_ (11.2 Ω) < NiPc (31.1 Ω) < NiO (85.8 Ω). This trend clearly indicates that [Ni_2_(tmd)_2_]_n_‐CV exhibits the most favorable charge‐transfer kinetics, further confirming its superior electrocatalytic activity for alkaline OER.

Following the methodology outlined by Lazanas et al., we performed EIS measurements on the [Ni_2_(tmd)_2_]_n_‐CV electrode under a series of applied potentials to gain deeper insight into the electrocatalytic reaction kinetics [47]. The potential was varied from 1.32 to 1.62 V vs. RHE in 0.06 V increments. Figure S14 presents the corresponding Bode plot, illustrating the relationship between applied potential (E), phase angle (φ), and the logarithm of frequency (log(f)). In the log(f) range of −1.5–1.5, a red region, indicating a high phase angle, extends from 1.32 to 1.45 V. This behavior suggests a non‐uniform charge distribution across the [Ni_2_(tmd)_2_]_n_‐CV surface. Specifically, within this potential window, the catalyst likely undergoes slower processes such as surface oxidation or limited mass transport of reactants from the electrolyte. This observation implies that the electrode experiences surface reconstruction or evolution prior to the onset of effective OER activity. This interpretation aligns well with the LSV results in Figure 5a, where there is no substantial increase in current density below 1.45 V, likely due to charge redistribution and incomplete activation of the catalyst. In the higher‐frequency region (log(f) = 1.0 to 2.5), which is primarily associated with charge‐transfer resistance at the electrode/electrolyte interface, a distinct shift in phase angle from red to green is observed as the potential surpasses 1.45 V. This transition indicates a significant improvement in charge‐transfer kinetics. These results suggest that once the applied potential exceeds 1.45 V, the dominant process becomes the OER driven by the adsorption of surface hydroxyl species and subsequent formation of Ni–OOH intermediates [48]. This inference is further supported by the LSV in Figure 5a, where the onset of rapid current increase occurs around 1.45 V. At higher potentials, the phase angle values in the same log(f) range stabilize (remaining green), confirming that OER, rather than additional material oxidation, is the primary electrochemical process taking place.

The electrochemically active surface area (ECSA) is an indicator of the double‐layer capacitance (C_dl_) on the electrode surface, which reflects the density of electrochemically active sites. Generally, a greater number of active sites leads to a higher C_dl_ [49]. Figure S15 presents the cyclic voltammetry (CV) curves of [Ni_2_(tmd)_2_]_n_‐CV, NiPc, RuO_2_, and NiO at various scan rates. The relationship between half of ΔI_OCP_ and the scan rate are plotted in Figure 5e. The C_dl_ values for each electrode were determined from the linear regression slopes, and the corresponding ECSAs were calculated using Equation (3) in the Experimental Section [50, 51]. The calculated ECSA values follow the order: NiO (0.925 cm^2^) < NiPc (4.7 cm^2^) < RuO_2_ (9.025 cm^2^) < [Ni_2_(tmd)_2_]_n_‐CV (13.2 cm^2^), indicating that [Ni_2_(tmd)_2_]_n_‐CV possesses the largest number of available electrochemical reaction sites. Figure 5f and Figure S16 summarize the durability tests of the different electrocatalysts. In Figure 5f, a constant potential of 1.45 V was applied, initiating the test at an initial current density of 10 mA/cm^2^. After 100 h of continuous operation, [Ni_2_(tmd)_2_]_n_‐CV retained approximately 93.9% of its initial current density, showing only a 6.1% drop and demonstrating excellent long‐term stability. In Figure S16, durability tests at a higher current density of 50 mA/cm^2^ were carried out by applying constant potentials of 1.63, 1.78, 1.69, and 2.02 V for [Ni_2_(tmd)_2_]_n_‐CV, NiPc, RuO_2_, and NiO, respectively. After 24 h, [Ni_2_(tmd)_2_]_n_‐CV maintained 86% of its initial current density, significantly outperforming NiPc (8.6%), RuO_2_ (39.0%), and NiO (9.0%). Figure S17 displays the HAADF‐STEM image of [Ni_2_(tmd)_2_]_n_‐CV after the 24 h durability test, along with the corresponding elemental maps for N, O, and Ni. The elemental distribution indicates that the composition of [Ni_2_(tmd)_2_]_n_‐CV remains stable, confirming its structural integrity during prolonged operation under OER conditions. Table S8 highlights the changes in STEM‐EDS elemental ratios following the durability test, indicating that the nitrogen‐containing ligand framework remains effectively coordinated to the metal centers despite minor surface‐limited metal dissolution and the potential formation of Ni–OH active species. In Figure S18, the XRD pattern of [Ni_2_(tmd)_2_]_n_‐CV after the stability test indicates a transition to an amorphous phase. The absence of detectable crystalline Ni oxide or hydroxide phases suggests that the structural evolution does not involve framework decomposition, confirming that the ligand environment is effectively preserved. Figure S19a present the Ni K‐edge XANES spectra of [Ni_2_(tmd)_2_]_n_‐CV and that after a 50 h stability test at the current density of 50 mA/cm^2^. The absorption edge shifts positively by approximately 0.4 eV and the white‐line intensity appears slightly higher after the durability test, indicating a partial oxidation of Ni species (Figure S19b). Figure S20 shows the corresponding FT‐EXAFS spectra for Figure S19a, and Table S9 present the fitting result and parameters for [Ni_2_(tmd)_2_]_n_‐CV after the durability test. From the fitting results, the primary Ni─N/O bond length remains nearly unchanged after long‐term operation. The coordination number of Ni–O slightly increases from 2.031 for [Ni_2_(tmd)_2_]_n_‐CV to 2.138, whereas those of Ni–N(S) and Ni–N(L) decrease marginally. These observations suggest that a small fraction of Ni─N bonds are substituted or reorganized in the OER process due to interaction with the intermediates or oxygen‐containing species in the electrolyte.

### 2.6 In Situ XAS Measurements for Alkaline OER Catalyzed by [Ni_2_(tmd)_2_]_n_‐CV

**FIGURE 6:**
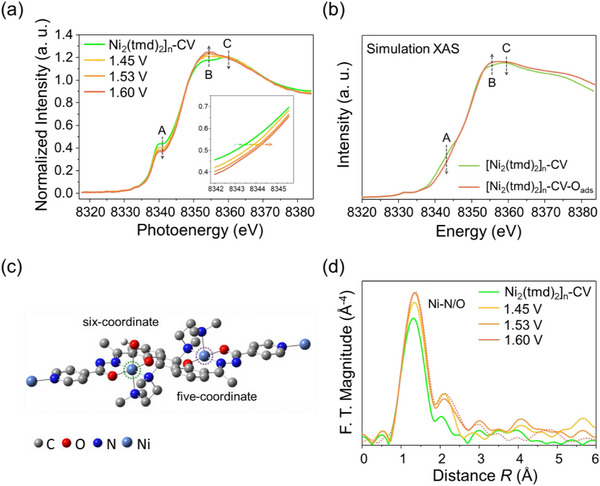
(a) Ni K‐edge XANES spectra of [Ni_2_(tmd)_2_]_n_ collected under different applied potentials during cyclic voltammetry (CV). The inset shows the enlarged pre‐edge region. (b) Simulated XANES spectra of [Ni_2_(tmd)_2_]_n_ and [Ni_2_(tmd)_2_]_n_​‐OH calculated by FDMNES. (c) Optimized structure of [Ni_2_(tmd)_2_]_n_ used for the FDMNES simulations. (d) Fourier‐transformed EXAFS spectra of [Ni_2_(tmd)_2_]_n_ under different applied potentials.

Although the electrochemical behavior and electronic structure of [Ni_2_(tmd)_2_]_n_ at various stages of the alkaline OER process have been characterized, real‐time information remains essential for fully elucidating the reaction mechanism. To this end, in situ X‐ray absorption spectroscopy (XAS) was employed to investigate the potential‐dependent changes in the electronic structure and local coordination environment of [Ni_2_(tmd)_2_]_n_‐CV during alkaline OER. All in situ XAS measurements were conducted in 1 M Fe‐reduced KOH (pH 13.8 ± 0.1), using an Hg/Hg_2_Cl_2_ electrode as the reference electrode (RE) and a graphite rod as the counter electrode (CE). A custom‐designed Teflon electrochemical cell equipped with a Kapton‐sealed window was used as the electrolyzer [52]. Figure 6a presents the Ni K‐edge XANES spectra of [Ni_2_(tmd)_2_]_n_‐CV recorded under open‐circuit potential (OCP) and applied potentials of 1.45, 1.53, and 1.60 V. Upon applying a potential of 1.45 V, a decrease in the intensity of peak A, an increase in peak B, and a decrease in peak C were observed. These spectral changes became more pronounced as the potential was increased. For [Ni_2_(tmd)_2_]_n_‐CV, the initial intensity ratio of B to C (I_B_/I_C_) was 0.975. With the elevated applied potential from 1.45 to 1.60 V, feature A progressively diminished, while the I_B_/I_C_ ratio increased to 1.01, 1.029, and 1.041, respectively. According to the study by Jia et al. on Fe–N_4_ planar molecules with D_4h_ symmetry, when the Fe atom is displaced along the *Z*‐axis from the planar center, the resulting distortion lowers the symmetry, and thereby reducing the contribution from the 1s → 4p_z_ transition (peak A) and enhancing that from the 1s → 4p_x_(4p_y_) transition. This effect leads to a decrease in the intensity of peak B, while peak C is influenced by the changes in the spin state or Fe─N bond length [53]. Given by this clue, we infer that during the oxygen evolution reaction (OER), the adsorption of intermediates may induce local structural distortions in [Ni_2_(tmd)_2_]_n_‐CV which leads to the variations in the peak intensities of A, B, and C.

To validate this estimation, FDMNES simulations were performed to model the change in the XANES spectra of [Ni_2_(tmd)_2_]_n_‐CV (Figure 4c,d) with adsorbed OER intermediates at Ni sites. As shown in Figure 6b,c, and Figure S21, when an oxygen atom is placed on one of the binuclear Ni sites at a distance of 2.6 Å (Figure 6c), the simulated XANES spectrum of [Ni_2_(tmd)_2_]_n_‐CV exhibits a weaker peak A, a stronger peak B, and a diminished peak C with the I_B_/I_C_ ratio increasing from 0.971 to 0.99 ([Ni_2_(tmd)_2_]_n_‐CV‐O_ads_ in Figure 6b). This variation can be primarily attributed to the redistribution of the 4p_z_ orbital electron density. Furthermore, to confirm the coordination and bond length variations, FT‐EXAFS spectra obtained under different potentials are presented in Figure 6d. Both the Ni─N/O bond length (R) and the corresponding peak intensity gradually increase from OCP to 1.53 V. Based on the simulated model, EXAFS fitting was performed by considering Ni–O, Ni–N(S), and Ni–N(L) coordination shells, as well as additional adsorbed oxygen (O_ads_) (Figure S22 and Table S9). The fitting results reveal that the coordination numbers of Ni–O, Ni–N(S), and Ni–N(L) remain nearly constant with increasing applied potentials, whereas that of Ni–O_ads_ increases from 0.56 to 0.71. It indicates a higher surface coverage of adsorbed oxygen species as the reaction kinetics accelerate, consistent with the variations of peak B and C observed in Figure 6a, and reflects local structural rearrangements induced by oxygen adsorption. On the other hand, the elevated intensity of the Ni–N/O peak (Figure 6d) may arise from the deprotonation of adsorbed oxygen species, leading to the formation of OOH intermediates – the key species involved in the OER pathway [54].

Different coordination geometries, such as octahedral, tetrahedral, and square‐planar structures, alter the symmetry and strength of the ligand field surrounding the metal center, leading to variations in the splitting pattern and magnitude (Δ) of the d orbitals. These changes in ligand‐field splitting affect the electronic configuration and spin arrangement, and thereby determining whether the complex exhibits a high‐spin or low‐spin state [55]. The Kβ_1,3_ emission line in X‐ray emission spectroscopy (XES) serves as a sensitive probe for distinguishing the spin state of Ni. This line originates from the 3p → 1s electronic transition, and its energy position is influenced by the exchange interaction between the 3p orbital and unpaired 3d electrons [56]. The Kβ' satellite feature in X‐ray emission spectroscopy (XES) originates from the exchange interaction between the 3p core hole and the unpaired 3d electrons in the final state. In a high‐spin state, the presence of a greater number of unpaired 3d electrons enhances this 3p–3d spin coupling, resulting in a larger exchange splitting that increases the intensity of the Kβ' satellite and shifts its position relative to the Kβ_1,3_ main line. Conversely, a transition to a low‐spin state (fewer unpaired electrons) weakens this interaction, leading to a diminished Kβ' feature. As shown in Figure S23a, the pristine [Ni_2_(tmd)_2_]_n_ (black) exhibits a distinct Kβ_1,3_ main peak, indicating that Ni^2^^+^ is in a low‐spin state. After KOH activation and CV treatment (red), the emergence of a Kβ' satellite feature on the low‐energy side reveals a transition of Ni^2^^+^ to a high‐spin state. Upon applying a potential of 1.45 V vs. RHE, the Kβ' intensity decreases, suggesting that oxygen adsorption at certain Ni sites induces a partial transformation to an octahedral coordination environment, and thus reverts the Ni^2^^+^ ions back to a low‐spin configuration. Figure S23b illustrates the ligand‐field splitting patterns of Ni^2^^+^ under different coordination geometries. The transition from an octahedral to a square pyramid structure reduces the ligand‐field splitting, leading to a high‐spin configuration of Ni^2^^+^. Conversely, adsorption of oxygen species restores the octahedral coordination, increasing the splitting energy and driving Ni^2^^+^ back into a low‐spin state.

### 2.7 In Situ Raman Measurements for Alkaline OER catalyzed by [Ni_2_(tmd)_2_]_n_‐CV

**FIGURE 7:**
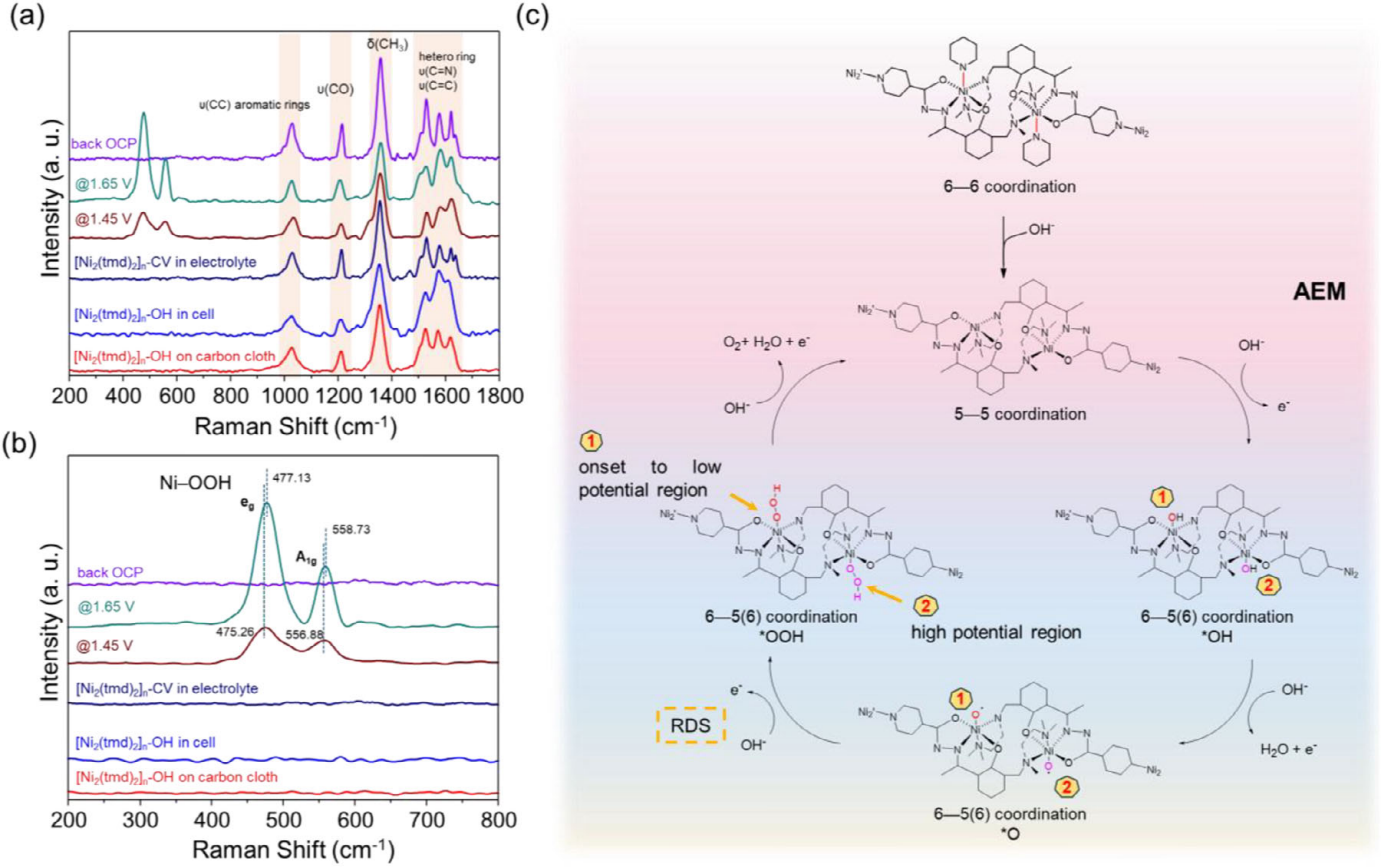
(a) Potential‐dependent in situ Raman spectra of [Ni_2_(tmd)_2_]_n_ recorded under various applied potentials. (b) Magnified view of the low‐frequency region (200–800 cm^−1^) from (a), highlighting the emergence of characteristic Ni‐OOH intermediate signals during the OER process. (c) Schematic illustration of the structural evolution and the proposed adsorbate evolution mechanism (AEM) for [Ni_2_(tmd)_2_]_n_ under alkaline conditions.

To gain deeper insights into the surface evolution of [Ni_2_(tmd)_2_]_n_‐CV during the oxygen evolution reaction (OER), in situ Raman spectroscopy was performed. Figure 7a,b displays the Raman spectra of [Ni_2_(tmd)_2_]_n_‐OH at various operational stages. The peaks observed in the 200–1000 cm^−1^ region are attributed to the characteristic vibrations of the organic ligand framework. Upon immersing the electrode into the quartz‐windowed cell and introducing the 1 M Fe‐reduce KOH electrolyte, Raman spectra were collected once the open‐circuit potential (OCP) stabilized; notably, no characteristic Ni–O signals were detected in the 200–1000 cm^−1^ range at this stage. Generally, Ni–OOH intermediates exist as either β‐Ni–OOH or γ‐Ni–OOH phases, with the latter often facilitating OER due to its relatively disordered structure and higher Nickel oxidation state [57, 58]. Upon applying a potential of 1.45 V(vs RHE), two distinct bands emerged at 475.26 and 556.88 cm^−1^, corresponding to the e_g_ bending and A_1g_ stretching modes of γ‐Ni–OOH, respectively [59]. Further increasing the applied potential resulted in a noticeable blueshift of both the e_g_ and A_1g_ signals, signifying the transition of Nickel centers to higher oxidation states [60]. Conversely, the signals associated with the organic framework remained constant throughout the potential application. This observation confirms that OH^−^ ions do not displace the H_2_tmd ligand during the OER process, demonstrating the exceptional structural stability of the coordination environment. Given by all the information, we could propose the structural transformation and the OER mechanism of [Ni_2_(tmd)_2_]_n_ as shown in Figure 7c. Initially, each Ni^2^^+^ center of [Ni_2_(tmd)_2_]_n_ (I, the pre‐catalyst) adopts an octahedral geometry coordinated by O and N atoms from tmd^2^^−^ ligands and amine nitrogen atoms from neighboring ligands, forming [Ni_2_(tmd)_2_] units. Upon immersion in the alkaline electrolyte (KOH), XANES analysis and corresponding simulations reveal that the Ni─N bond associated with the pyridine moiety preferentially breaks, leading to a five‐coordinate square pyramid configuration (II, the activated catalyst). Although the Ni–N (pyridine) bond is not the longest among the six Ni─N/O bonds, it is the first to dissociate due to the local coordination environment of the Ni center. Specifically, each Ni center adopts an octahedral coordination geometry, being coordinated by a tridentate pocket from one tmd^2^^−^ ligand, a bidentate pocket from a neighboring tmd^2^^−^ ligand, and only one pyridine nitrogen atom from a third ligand. This coordination arrangement also provides a structural rationale for the remarkable stability of [Ni_2_(tmd)_2_]_n_ over more than 100 catalytic cycles, as dissociation of the monodentate pyridine N donor does not disrupt the dinuclear Ni_2_ core. The Tafel slope (94.15 mV dec^−1^), in situ Raman spectroscopy, and in situ XAS results confirm that the OER primarily follows the surface adsorbate evolution mechanism (AEM), where product formation proceeds through surface‐adsorbed intermediates that evolve sequentially on the catalyst surface [61, 62]. Specifically, as evidenced by XANES variations and XAS simulations, the adsorbed oxygen species (O^*^) preferentially coordinate with one Ni center within the dinuclear unit during the OER process. At more elevated potentials, O^*^ species subsequently occupy the second Ni site, allowing the reaction to proceed via the conventional four‐electron transfer pathway″.

### 2.8 Density Functional Theory Calculations

**FIGURE 8:**
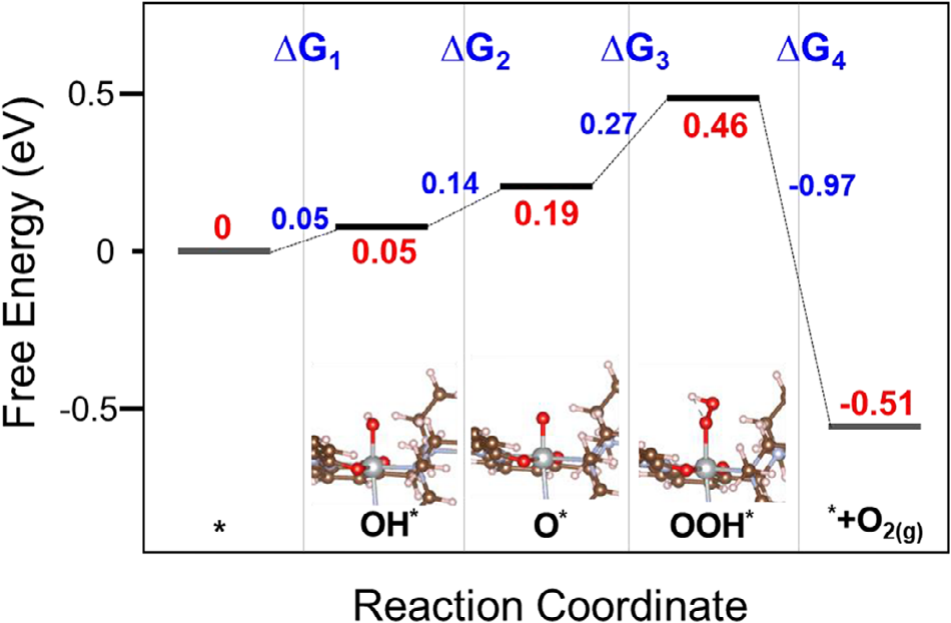
DFT‐calculated free energy diagram and corresponding structures for the OER over [Ni_2_(tmd)_2_]_n_ via the AEM pathway. The Gray, brown, red, blue, and pink spheres represent Ni, C, O, N, and H atoms, respectively.

Density functional theory (DFT) calculations were performed to elucidate the variations in alkaline OER over [Ni_2_(tmd)_2_]_n_. The calculated OER free energy profiles, together with the corresponding structures of the key reaction intermediates (^*^OH, ^*^O, and ^*^OOH) on the active sites, are presented in Figure 8. The DFT results indicate that the potential‐determining step (PDS) for the OER over [Ni_2_(tmd)_2_]_n_ following the proposed adsorbate evolution mechanism (AEM) occurs at the third elementary step (O^*^ + OH^−^ → OOH^*^ + e^−^, ΔG3). This step exhibits a free energy change of 0.27 eV, corresponding to a low theoretical overpotential and showing good qualitative agreement with experimental trends.

## 3 Conclusion

Designing pre‐catalyst structures that evolve into highly active and durable catalysts upon activation is a key strategy for advancing next‐generation catalysis. Through rational design of the Schiff base ligand H_2_tmd, we synthesized a novel nickel‐based coordination polymer (CP) featuring a mirror‐symmetry dinuclear Ni–Schiff base coordination network. The H_2_tmd ligand, offering an O, N, N, O coordination pocket, enables the formation of a six‐coordinate complex, [Ni_2_(tmd)_2_]_n_, where each Ni center is coordinated by two Ni─O and four Ni─N bonds. In alkaline conditions, [Ni_2_(tmd)_2_]_n_ acts as a pre‐catalyst that undergoes structural transformation in 1 M Fe‐depleted KOH to form [Ni_2_(tmd)_2_]_n_‐OH. This transformation involves the loss of coordination with the dimethylamino and pyridinyl nitrogen atoms, resulting in a square pyramid five‐coordinate Ni environment. The [Ni_2_(tmd)_2_]_n_‐OH exhibits excellent structural stability and enhanced surface activity during repeated cyclic voltammetry (CV) scans, ultimately yielding the catalytically active form, [Ni_2_(tmd)_2_]_n_‐CV. The activated [Ni_2_(tmd)_2_]_n_‐CV demonstrated superior electrocatalytic performance in alkaline OER, with low overpotentials (η_10_ = 222 mV and η_50_ = 385 mV), a small Tafel slope (94.15 mV/dec), and quite low charge‐transfer resistance (R_ct_ = 3.1 Ω), all outperforming those of benchmark catalysts including NiPc, RuO_2_, and NiO. Moreover, [Ni_2_(tmd)_2_]_n_‐CV maintained excellent durability, retaining 93.9% of its initial current density after 100 h of continuous operation. In situ XAS analysis, supported by detailed spectral fitting, revealed potential‐dependent changes in the Ni K‐edge XANES and EXAFS spectra. These changes are attributed to adsorption of oxygen species at the five‐coordinate Ni sites, likely associated with the formation of Ni–OOH species, which are critical for OER activity. To our best knowledge, this study provides the first direct evidence of a transformation from six‐ to five‐coordinate Ni centers in a dinuclear coordination polymer, leading to an efficient and stable OER catalyst. Our strategies for the pre‐catalyst design and characterization are broadly applicable to other coordination polymers, offering significant potential and interest in the field of electrocatalysis [63, 64, 65, 66, 67, 68, 69, 70, 71, 72].

## Conflicts of Interest

The authors declare no conflicts of interest.

## Supporting information




**Supporting File**: advs74174‐sup‐0001‐SuppMat.docx.

## Data Availability

The data that support the findings of this study are available from the corresponding author upon reasonable request.
