# Supplementary material for: Harnessing Transformation of Metal‐Ligand Coordination in Dinuclear Ni(II)‐Schiff Base Coordination Polymer for Promoting Electrochemical Oxygen Evolution
Source: Adv Sci (Weinh). 2026 Jan 29;13(20):e24014. doi: 10.1002/advs.202524014 (PMC13067823; doi:10.1002/advs.202524014)
Supplement: Supplementary file 1 — Supporting File: advs74174‐sup‐0001‐SuppMat.docx. [file ADVS-13-e24014-s001.docx]

Supporting Information

**Harnessing Transformation of Metal-Ligand Coordination in Dinuclear Ni(II)-Schiff Base Coordination Polymer for Promoting Electrochemical Oxygen Evolution**

Ruei-Hung Juang,^[a]^ Han-Jung Li,^[b]^ Yu-Chung Chang,^[c]^ Chieh-Wei Chang,^[d]^ Ricky Yu-Syun Fan,^[e]^ Kien Voon Kong,^[e]^ Jeng-Lung Chen,^[f]^ Chia-Shuo Hsu,*^[f]^ Po-Heng, Lin,*^[d]^ Chun-Hong Kuo*^[a]^

[a] Department of Applied Chemistry, National Yang Ming Chiao Tung University, Hsinchu 300093, Taiwan

Email: chunhong@nycu.edu.tw

[b] Department of Chemistry, Chung Yuan Christian University, Taoyuan 320314, Taiwan
E-mail: a896420127@gmail.com

[c] Department of Materials Science and Engineering, Feng Chia University, Taichung 407102, Taiwan
E-mail: ychungchang@o365.fcu.edu.tw

[d] Department of Chemistry, National Chung Hsing University, Taichung 402202, Taiwan

Email: poheng@dragon.nchu.edu.tw

[e] Department of Chemistry, National Taiwan University, Taipei 106319, Taiwan
E-mail: kvkong@ntu.edu.tw

[f] National Synchrotron Radiation Research Center, Hsinchu 300092, Taiwan

Email: hsu.chiashuo@nsrrc.org.tw

**EXPERIMENTAL SECTION**

**Chemicals.** Paraformaldehyde (97%, Alfa Aesar), N, N, N'-trimethylethylenediamine (97%, Alfa Aesar), 2'-hydroxyacetophenone (98%, Alfa Aesar), toluene (≥ 99.3%, Avantor), dichloromethane (DCM, 99.9%, Duksan Pure Chemicals), magnesium sulfate anhydrous (MgSO_4_, 99.5%, Showa Chemical), hexane (95%, UniRegion Bio-Tech), ethyl acetate (99.9%, Duksan Pure Chemicals), ethyl ether (≥ 99%, Merck), methanol (MeOH, 99.8%, Duksan Pure Chemicals), ethanol (EtOH, 99.5%, ECHO chemical), isonicotinic acid hydrazide (≥ 98%, Alfa Aesar), triethylamine (NEt_3_, ≥ 99%, J. T. Baker). nickle(II) nitrate hexahydrate (Ni(NO_3_)_2_∙6H_2_O, ≥ 98%, Sigma-Aldrich), ruthenium oxide (RuO_2_, ~ 200 mesh, 99.9%, Alfa Aesar), nickel oxide (NiO, ~320 mesh, 99%, Alfa Aesar), nickel phthalocyanine (NiPc, ≥ 95%, Alfa Aesar), potassium hydroxide (KOH, ≥ 85%, Sigma-Aldrich), carbon paper (0.18 mm thick, CeTech), Nafion-perfluorinated resin solution (5 wt% in lower aliphatic alcohols and water, containing 15‒20% water, Sigma-Aldrich). All chemicals and solvents were used as received without further purification. Ultrapure deionized water (DI H_2_O, 18.2 MΩ·cm) was used to prepare all solutions for electrochemical experiments.

**Synthesis of Ligand H_2_tmd.** H_2_tmd is the abbreviation for the ligand of (E)-N'-(1-(3-(((2-(dimethylamino)ethyl)(methyl)amino)methyl)-2-hydroxyphenyl)ethylidene)isonicotinohydrazide. The representative chemical equations for the synthesis of H_2_tmd is shown in **Scheme S1**. In the synthesis, a flask containing paraformaldehyde (110 mmol, 3.3033g) was prepared, and N, N, N'-trimethylethylenediamine (100 mmol, 13 mL) and 2'-hydroxyacetophenone (110 mmol, 13.3 mL) in toluene (250 mL) were added and refluxed for 2 days. The resulting brown solution was diluted with DCM and washed with DI H_2_O. After the separation, the organic layer was dried over anhydrous MgSO_4_. The DCM fraction was subjected to column chromatography (CC) on silica gel. The column was first eluted with the mixed solution of hexane and ethyl acetate (v/v = 8/1) and then with that of DCM and MeOH (v/v = 6/1). The fraction obtained in DCM/MeOH (v/v = 6/1) was subjected to column chromatography to afford 1-[3-[[[2-(dimethylamino)ethyl]methylamino]methyl]-2-hydroxyphenyl]ethanone (14.397 g). The product was dried in vacuum to yield a yellow oily liquid (yield: 57%). 1H-NMR (CD_3_OD, ppm): 7.83 (d, 1H), 7.51 (d, 1H), 6.92 (t, 1H), 3.64 (s, 2H), 2.65 (s, 3H), 2.58 (d, 4H), 2.25 (s, 6H). With 1-[3-[[[2-(dimethylamino)ethyl]methylamino]methyl]-2-hydroxyphenyl]ethanone (57.5 mmol, 14.397 g) in the flask, isonicotinic acid hydrazide (57.5mmol, 7.8855 g) and MeOH (200 mL) were added and refluxed for 1 day. The concentrated solution was dried under vacuum and washed three times with DCM, followed by the addition of ethyl acetate. It was dried again in a vacuum, leading to a pale-yellow precipitate. The resulting product was then filtered along with washing by using ethyl acetate and ether, and eventually dried in a vacuum system to obtain 14.8712 g of H_2_-tmd (yield: 70%). 1H-NMR (CD_3_OD, ppm): 8.59 (d, 2H), 8.06 (d, 2H), 7.54 (d, 1H), 7.15 (d, 1H), 6.55 (t, 1H), 3.45 (s, 2H), 3.36 (t, 2H), 2.98 (s, 6H), 2.80 (t, 2H), 2.77 (s, 3H), 2.25 (s, 3H).

**Synthesis of** **Complex** **[Ni_2_(tmd)_2_]_n_.** In the typical synthesis, a solution of Ni(NO_3_)_2_·6H_2_O (0.25 mmol, 0.0456 g) in MeOH (15 mL) was added into that of H_2_tmd (0.25 mmol, 0.0924 g), and triethylamine (1.25 mmol, 0.174 mL) in DCM (15 mL). The mixed solution (orange color) was stirred for 1 min and filtered. Next, it was settled for 1 day and resulted in the orange crystals of [Ni_2_(tmd)_2_]_n_ that were then collected by filtration, washed with ether, and dried in vacuo. [Ni_2_(tmd)_2_]_n_: Yield = 38%. IR (KBr, cm^-1^): 1735 (w), 1719 (w), 1701 (w), 1685 (w), 1654 (m), 1637 (w), 1618 (m), 1594 (s), 1578 (s), 1560 (m), 1541 (s), 1535 (s), 1508 (s), 1459 (s), 1448 (m), 1420 (s), 1363 (m), 1251 (s), 1016 (w), 748 (w); Anal. calcd. for C_40.5_H_51_N_10_Ni_2_O_4_Cl ([Ni_2_(tmd)_2_]_n_·0.5 CH_2_Cl_2_): C, 54.22; H, 5.56; N, 15.49. Found: C, 54.37; H, 5.75; N, 15.65.

**Characterization.** Single crystals of [Ni_2_(tmd)_2_]_n_ suitable for X-ray diffraction measurements were mounted on an Oxford Xcalibur Sapphire-3 CCD Gemini diffractometer employing graphite-monochromated MoKα radiation (λ = 0.71073 Å), and intensity data were collected with ω scans. The data collection and reduction were performed with the CrysAlisPro software, and the absorptions were corrected by the SCALE3 ABSPACK multi-scan method. The space-group determination was based on a check of the Laue symmetry and systematic absences, and it was confirmed using the structure solution. The structure was solved and refined with the *Olex2 1.5* package. Anisotropic thermal parameters were used for all non-H atoms, and fixed isotropic parameters were used for H atoms. All samples for transmission electron microscopy (TEM) were made by dispersing catalyst in a mixed solution of H_2_O/EtOH and then dropping onto lacey copper grids coated with carbon films. Bright-field (BF) images, high-angle annular dark-field scanning transmission electron microscopy (HAADF-STEM) images, and energy-dispersive X-ray spectroscopy (EDS) were done using a JEOL JEM-ARM200FTH TEM equipped with a spherical aberration corrector (Cs-corrected) at an accelerating voltage of 200 kV. The metal content of the electrolyte and electrodes was analyzed by inductively coupled plasma mass spectrometry (ICP-MS) using a PerkinElmer NexION 350, after digestion of the samples in ultrapure nitric acid and dilution to appropriate concentrations. High-resolution powder X-ray diffraction (HRPXRD) data were acquired at the Taiwan Photon Source (TPS 19A beamline) in the National Synchrotron Radiation Research Center (NSRRC) in Hsinchu City, Taiwan, with an X-ray energy of 20 keV and a wavelength of 0.61992 Å. The samples were loaded into 0.3 mm quartz capillaries for measurements. Data analysis and refinement were performed using the GSAS-II software package.

**Preparation of Iron-Reduced KOH Electrolyte****.** To avoid the influence in electrocatalysis from the existing iron ions in DI H_2_O as much as possible, the preparation of the iron-reduced KOH electrolyte was carried out by referring to the former method with modifications.^[63]^ In a clean polypropylene centrifuge tube (50 mL), 3 g of Ni(NO_3_)_2_·6H_2_O was dissolved in 5 mL of DI H_2_O (18.2 Ω·cm), followed by adding 20 mL of 1 M KOH aqueous solution to form the light green Ni(OH)_2_ colloids. The mixture was centrifuged at 5000 rpm for 5 min (Eppendorf Centrifuge 5804), and the supernatant was decanted. The left precipitate was washed by rinsing with DI H_2_O, and the centrifuging-washing process was repeated three times. Afterwards, the cleaned Ni(OH)_2_ colloids were dispersed in 40 mL of 1 M KOH. Next, transferring 40 mL of 1 M KOH solution containing Ni(OH)_2_ to 250 mL of 1 M KOH with vigorous stirring overnight, and leaving it for one day to settle the colloids down. The concentrations of iron (Fe) and nickel (Ni) ions in the supernatant were measured by ICP-MS, giving that the concentrations of Fe and Ni ions were lower than 0.8 ppb and 2.5 ppb, respectively. We assumed that the influence in electrocatalysis from such low Fe concentration was ignorable, and thus the resulting supernatant solution, unless otherwise specified, was used as the electrolyte throughout all experiments in this work.

### Electrocatalytic Oxygen Evolution Reaction (OER) Measurements. All electrochemical measurements were done in a standard three-electrode system (CHI 760E) in the 1 M Fe-reduced KOH (pH = 13.8 ± 0.1). The catalyst was prepared as an ink by dispersing 5 mg of catalysts in a mixed solution of 45 µL DI H_2_O, 45 µL EtOH, and 10 µL of 0.5% Nafion solution and ultrasonic agitation for 30 minutes. To prepare the working electrode, the catalyst ink was drop-casted onto double sides of carbon paper (20 x 5 mm^2^/pc) and dried overnight in a vacuum oven at 45 °C. A graphite rod was used as the counter electrode, and a saturated calomel electrode (SCE, Hg/Hg_2_Cl_2_) served as the reference electrode. The values of measured potentials (vs SCE) in all experiments were calibrated to the reversible hydrogen electrode (RHE) following the equation as below.

### E_RHE_ = E_REF_(Hg/Hg_2_Cl_2_) + 0.059 × pH (1)

Prior to the OER LSV measurements, the catalysts were activated by performing 150 cycles of cyclic voltammetry (CV) in the potential range of 0 to 1.1 V (vs. Hg/Hg_2_Cl_2_) at a scan rate of 20 mV/s without iR compensation until a stable polarization curve was achieved. Subsequent LSV tests were then conducted with 85% iR compensation, with the solution resistance (R_s_) typically found to be approximately 5.5 Ω. Electrochemical impedance spectroscopy (EIS) measurements were performed by applying different potentials within the frequency range of 0.01 Hz to 10 kHz, with an amplitude of 0.005 V. The real part of the double-layer capacitance C'(ω) is described as follows,^[64]^

C'(ω) = $\frac{\text{C(ω)}}{\text{1+ }\text{R}^{\text{2}}\left( \text{ω} \right)\text{ × }\text{C}^{\text{2}}\left( \text{ω} \right)\text{ × }\text{ω}^{\text{2}}}$ = $\frac{\text{-Z''(ω)}}{{\text{ω}\left| \text{Z(ω)} \right|}^{\text{2}}}$ (2)

where R(ω) and C(ω) are the double-layer resistance and capacitance of the pulsation ω, Z'' is the imaginary part of the impedance, |Z(ω)| is electrochemical impedance, and ω is the angular frequency (ω = 2πf). The electrochemical double-layer capacitance (C_dl_) was evaluated by collecting cyclic voltammogram (CVs) in the potential range within a non-faradic process at different scan rates (*r*) of 20, 40, 60, 80, and 100 mV/s. The potential windows of ECSA were determined by the open-circuit potential (OCP) ± 0.05 V (vs RHE). Then, the double-layer capacitance (C_dl_), which is half of d(Δ*I*)/d(*v*), was estimated by plotting Δ*I*_OCP_ = (*I*_top_ − *I*_bottom_ at OCP) as a function of the scan rate (*v*). The electrochemical active surface areas (ECSAs) were estimated according to the following formula.

ECSA = C_dl_/C_s_ (3)

C_s_ is the specific capacitance of a flat surface with 1 cm^2^ of real surface area, which is generally in the range of 0.02 to 0.06 mF/cm^2^. Thus, the averaged value of 0.04 mF/cm^2^ was assumed for the flat electrode.

**In-Situ electrochemical Raman measurements.** In situ electrochemical Raman measurements were conducted using a custom-made electrochemical cell containing 1 M Fe-reduce KOH as the electrolyte. Raman spectra were acquired on a Renishaw inVia microprobe Raman spectrometer with a 532 nm excitation laser operated at 2.5 mW, covering a spectral range from 200 to 1800 cm⁻¹. An Ag/AgCl electrode and a platinum wire served as the reference and counter electrodes, respectively. Following electrochemical activation via cyclic voltammetry (CV) until a stable polarization curve was achieved, specific potentials were applied to the working electrode and maintained for 5 min prior to spectral collection to ensure steady-state conditions.

**Ex-Situ and In-Situ X-Ray Absorption and Emission Spectroscopy Measurements.** The measurements were done using quick-scanning X-ray absorption spectroscopy (XAS) at the TPS BL44A beamline in NSRRC in Hsinchu City, Taiwan. The XAS spectra for the Ni K-edges were recorded in the transmission mode, with a Ni metal foil employed for energy calibration. The energy resolution for the Ni K-edge, corresponding to a photon energy of 8333.0 eV, was set at 0.3 eV. XAS was used to investigate the local atomic environment around the absorbing Ni atoms in the catalysts, examining parameters such as coordination, structural disorder, and interatomic distances. The X-ray absorption near-edge structure (XANES) provided insights into the electronic and geometric configuration, while the extended X-ray absorption fine structure (EXAFS) delivered more detailed structural information, including the identity and quantity of neighboring atoms surrounding the absorbing centers. All XAS data were processed using the Demeter software suite, specifically utilizing Athena for data normalization and Artemis for fitting. In the analysis, N denotes the coordination number, R represents the distance between the absorber and scattering atoms, σ² is the Debye-Waller factor, and R_f_ reflects the goodness of fit. The uncertainties for the structural parameters derived from the EXAFS analysis were approximately ± 20% for N, ± 1% for R, and ± 20% for σ². To further explore the electrochemical catalytic behaviors of the samples, in-situ XAS measurements were carried out in 1 M Fe-reduced KOH electrolytes (pH = 13.8 ± 0.1). The in-situ XAS measurements were performed using a standard three-electrode setup, with carbon paper as the working electrode, an SCE reference electrode, and a graphite rod as the counter electrode. The setup was housed within a custom-designed Teflon cell, featuring a Kapton-tape-sealed window.^[52]^ After a 10-minute stabilization period, the in-situ XAS spectra were collected. X-ray emission spectroscopy (XES) was performed at the beamline 12XU in Spring-8, Japan. The incident beam energy, which was constant at 8363 eV for Ni Kβ, was monochromatized by a Si (111) double-crystal monochromator. The monochromatized beam was allowed to transmit through the tape and electrolyte at an incident angle of 0˚. The fluorescence was split by the analyzer crystal Si (444), with signals collected by a silicon drift detector (XR-100CR Si-PIN X-ray detector) in the scan range of 8235–8285 eV for Fe Kβ in BL-12XU at Spring-8.

**XAS simulation.** Ni K-edge X-ray absorption spectra (XAS) were simulated using the Finite Difference Method Near Edge Structure (FDMNES) code, which is based on ab initio density functional theory (DFT) calculations.^[65]^ A self-consistent calculation of the scattering potential around the nickel absorber was performed within a 6 Å cluster radius. To achieve optimal agreement with experimental spectra, a fully screened grid was implemented, and the finite difference method (FDM) was applied. The input CIF files for [Ni_2_(tmd)_2_]_n_ were generated from single-crystal X-ray diffraction results. To model the solvated complex [Ni_2_(tmd)_2_]_n_-OH, the CIF structure was modified by removing the ligands along the Z-axis, representing the structural change upon dissolution. Conversely, the [Ni_2_(tmd)_2_]_n_-CV model was constructed based on the [Ni_2_(tmd)_2_]_n_-OH structure, where additional OH groups were introduced along the Z-axis to mimic the coordination environment under OER conditions.

**Density Functional Theory Calculations.** All calculations were performed using the DFT plane-wave method as implemented in the Vienna Ab initio Simulation Package (VASP). The projector-augmented-wave (PAW) pseudopotentials were employed with an energy cutoff of 400 eV, together with the Perdew–Burke–Ernzerhof (PBE) exchange–correlation functional.^[66, 67, 68,69]^ A 25 × 25 × 25 Å³ cubic supercell was adopted to ensure sufficient vacuum separation and convergence of the total energy. Brillouin zone integrations were carried out using the Γ-point sampling.^[70]^

The oxygen evolution reaction (OER) was modeled at the active sites (*) based on the four-electron mechanism proposed by Nørskov.^[71, 72]^ The reaction steps are as follows:

OH^−^ + * → OH* + e^−^ ∆G_1_ (1)

OH* → O* + H^+^ + e^−^ ∆G_2_ (2)

O* + OH^−^ → OOH* + e^−^ ∆G_3_ (3)

OOH^*^ → O_2_ + * + H^+^ + e^−^ ∆G_4_ (4)

where the *, OH*, O*, and OOH^*^ represent the pure surface, adsorbed OH, O, and OOH species, respectively. Then, the free energy, ΔG, was calculated according to the following equation:

ΔG = ΔE + ΔE_ZPE_ − TΔS (5)

where ΔE, ΔE_ZPE_, and ΔS represent the changes of electronic energy, zero-point energy, and entropy, respectively.

***Scheme S1*.** The chemical equations for the two-step synthesis of H_2_tmd.

***Scheme S2*.** The potential coordination modes of H_2_tmd ligands with 3d and 4f metals.

***Table S1.*** Selected bonds (Å) and angles (°) for dinuclear Ni-based coordination polymer.

| **Bond Lengths (Å)** | | | |
| --- | --- | --- | --- |
| Ni1 | | Ni2 | |
| Ni1-O1 | 1.980(4) | Ni2-O3 | 2.011(4) |
| Ni1-O2 | 2.027(4) | Ni2-O4 | 1.957(4) |
| Ni1-N1' | 2.213(6) | Ni2-N6' | 2.250(6) |
| Ni1-N2' | 2.129(5) | Ni2-N7' | 2.153(5) |
| Ni1-N3 | 2.002(5) | Ni2-N8 | 2.003(6) |
| Ni1-N10 | 2.141(6) | Ni2-N5 | 2.195(5) |
| **Bond Angles (°)** | | | |
| Ni1 | | Ni2 | |
| N2'-Ni1-N1' | 83.9(2) | N7'-Ni2-N6' | 81.9(2) |
| O1-Ni1-N3 | 90.34(19) | O3-Ni2-N8 | 80.8(2) |
| N3-Ni1-O2 | 80.04(19) | N8-Ni2-O4 | 89.6(2) |
| O1-Ni1-N10 | 89.20(19) | O3-Ni2-N5 | 89.91(19) |
| N3-Ni1-N10 | 86.5(2) | N8-Ni2-N5 | 88.4(2) |
| N2'-Ni1-N10 | 91.7(2) | N7'-Ni2-N5 | 93.7(2) |

***Figure S1.*** Perspective views of the packing arrangements of [Ni_2_(tmd)_2_]_n_ viewed along the (a) a-axis, (b) b-axis, and (c) c-axis. Color code: green (Ni), red (O), blue (N) and grey (C). The hydrogen atoms and solvent molecules are omitted for clarity.

***Table S2.*** Crystallographic data of [Ni_2_(tmd)_2_]_n_ powder obtained from synchrotron PXRD.

| Formular | C_332_ H_424_ Cl_24_ N_80_ Ni_16_ O_32_ |
| --- | --- |
| Molecular weight (g/mol) | 1603.76 |
| Space group | Ibca (Orthorhombic) |
| λ (Å) | 0.61992 |
| 2θ(°) | 3 to 58.5 |
| a(Å) | 38.680(5) |
| b(Å) | 21.141(9) |
| c(Å) | 32.435(8) |
| V (Å^3^) | 21722.907 |
| Micro µ Equatorial strain | 4038.9 |
| Micro µ Axial strain | 2285.5 |
| Rwp | 4.291% |
| Zero point shift (°) | -0.005 |
| Uiso Ni (Å^2^ ) | 0.02120 |
| Uiso Ni (Å^2^ ) | 0.02110 |
| Uiso Ni (Å^2^ ) | 0.02290 |
| Uiso Ni (Å^2^ ) | 0.02310 |
| R(F^2^) | 4.640% |

***Figure S2.*** First derivative XANES of NiO and [Ni_2_(tmd)_2_]_n_.

***Table S3.*** Structural parameters from Ni K-edge EXAFS fitting for [Ni_2_(tmd)_2_]_n_, [Ni_2_(tmd)_2_]_n_-OH, [Ni_2_(tmd)_2_]_n_-OH after 150 CV cycles, NiPc, NiO, and Ni foil.

|  | path | R (Å) | N | △E (eV) | σ^2^(Å ^2^) | R factor |
| --- | --- | --- | --- | --- | --- | --- |
| [Ni_2_(tmd)_2_]_n_ | Ni-O | 2.006 | 1.98 | -0.109 | 0.005 | 0.008 |
|  | Ni-N(S) | 2.008 | 1.954 | -7.283 | 0.006 |  |
|  | Ni-N(L) | 2.207 | 2.028 |  |  |  |
|  | Ni-C | 2.958 | 4.306 | 2.032 | 0.008 |  |
| [Ni_2_(tmd)_2_]_n_-OH | Ni-O | 1.744 | 2.004 | −15.530 | 0.002 | 0.001 |
|  | Ni-N(S) | 1.946 | 2.053 | -9.829 | 0.003 |  |
|  | Ni-N(L) | 2.302 | 1.090 |  |  |  |
| 150th CV | Ni-O | 1.796 | 2.030 | -7.191 | 0.003 | 0.001 |
|  | Ni-N(S) | 1.954 | 2.032 | -9.037 | 0.002 |  |
|  | Ni-N(L) | 2.237 | 1.074 |  |  |  |
| NiPc | Ni-N | 1.910 | 4.066 | −13.213 | 0.012 | 0.008 |
|  | Ni-C | 3.012 | 3.614 | -8.954 | 0.010 |  |
| NiO | Ni-O | 2.083 | 6 | −2.426 | 0.009 | 0.005 |
|  | Ni-O-Ni | 2.953 | 12 | -3.483 | 0.008 |  |
| Ni foil | Ni-Ni | 2.487 | 12 | -7.106 | 0.007 | 0.008 |

Fitting was done across the *k* range of 2.25 to 9 Å^–1^ and the *R* range of 1.1 to 3 Å for [Ni_2_(tmd)_2_]_n_ and NiPc. *k* range of 2.25 to 9 Å^–1^ and the *R* range of 1.1 to 2.8 Å for [Ni_2_(tmd)_2_]_n_-OH, 150^th^ CV. *k* range of 2.25 to 11 Å^–1^ and the *R* range of 1.6 to 2.75 Å for Ni foil, *k* range of 2.25 to 11 Å^–1^ and the *R* range of 1.3 to 3 Å for NiO.

***Figure S3.*** Ni K-edge XANES spectra of [Ni_2_(tmd)_2_]_n_ powder after 1 M KOH treatment. The pre-edge feature at 8335 eV corresponds to the 1s → 4p_z_ transition, indicating changes in the electronic structure upon KOH treatment. The inset highlights the difference in the pre-edge feature.

***Figure S4.*** Ni K-edge EXAFS spectra for [Ni_2_(tmd)_2_]_n_, [Ni_2_(tmd)_2_]_n_-OH, NiPc, and NiO.


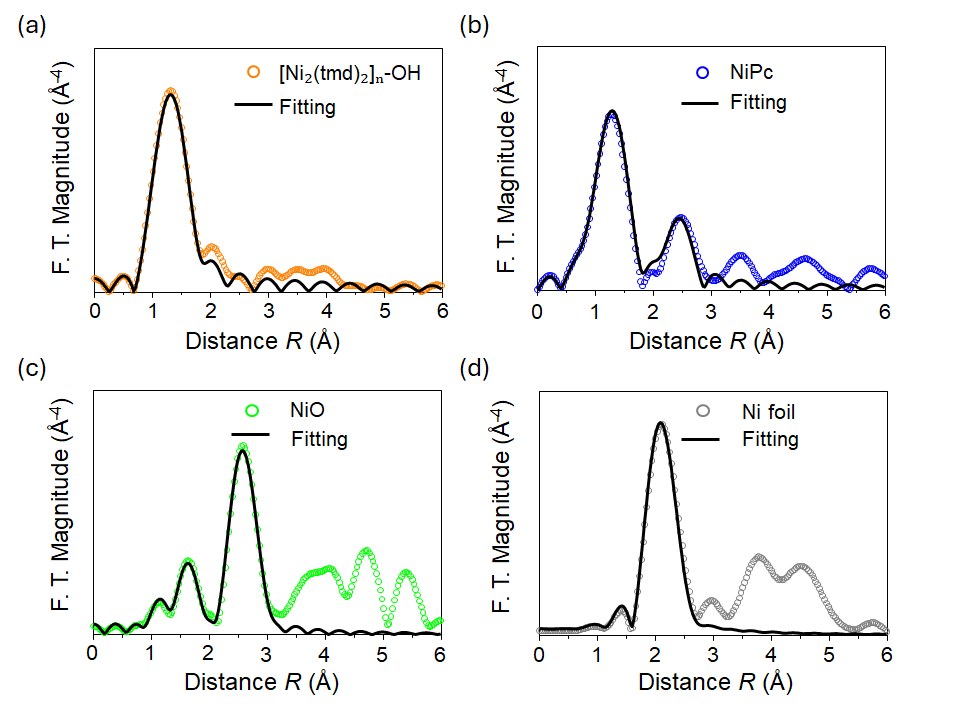


***Figure S5.*** Ni K-edge EXAFS fitting results for (a) [Ni_2_(tmd)_2_]_n_-OH, (b) NiPc, (c) NiO, and (d) Ni foil.

***Figure S6.*** (a) Cyclic voltammetry (CV) curves of [Ni_2_(tmd)_2_]_n_-OH in 1 M KOH solution through 150-cycle scans at a scan rate of 20 mV/s. The inset panel represents the enlarged region from 1.10 to 1.45 V, highlighting the redox peaks. (b) Tafel plots of [Ni_2_(tmd)_2_]_n_-OH at different CV cycles. The Tafel slopes were calculated for the 1st, 10th, 30th, 50th, 100th, and 150th CV cycles.

***Table S4.*** The values of open-circuit potentials, overpotential (η_10_), and Tafel slope for [Ni_2_(tmd)_2_]_n_-OH electrocatalyst at different CV cycles in 1 M KOH electrolyte.

| **CV Cycle** | **OCP (V)** | **η_10_ (mV)** | **Tafel Slope (mV/dec)** |
| --- | --- | --- | --- |
| 1st | 0.110 | N/A  (789 mV@6.63 mA/cm^2^) | 407.9 ± 14.5 |
| 10th | 0.048 | 661 ± 11 mV | 297.7 ± 22.8 |
| 30th | 0.060 | 435 ± 16 mV | 134.1 ± 13.1 |
| 50th | 0.140 | 365 ± 23 mV | 96.1 ± 7.2 |
| 100th | 0.150 | 351 ± 13 mV | 90.1 ± 3.7 |
| 150th | 0.150 | 330 ± 18 mV | 88.2 ± 4.2 |

***Figure S7.*** Electrochemical impedance spectroscopy (EIS) analysis after different CV cycles. (a) The Bode plot of phase difference vs log(f) where f denotes the frequency in EIS. (b) The schematic interpretation for the results in Figure S7a, the Faradaic process at the high-frequency region (e.g. log(f) = 4). (c) The plot showing the double-layer capacitance C'(ω) vs log(f). (d) The schematic illustration for the results in Figure S7c at the low-frequency region (e.g. log(f) = −2).

***Figure S8*.** The example of Ni K-edges of [Ni_2_(tmd)_2_]_n_ and [Ni_2_(tmd)_2_]_n_-OH shows the normalization by setting the absorption coefficients as 1.0.

***Figure S9.*** (a) Ni K-edge XANES spectra of [Ni_2_(tmd)_2_]_n_, [Ni_2_(tmd)_2_]_n_-OH, and the samples after CV scans for different cycles. (b) The enlarged area of A for [Ni_2_(tmd)_2_]_n_ and [Ni_2_(tmd)_2_]_n_-OH.

***Figure S10.*** Wavelet transforms of (a) [Ni_2_(tmd)_2_]_n_, (b) [Ni_2_(tmd)_2_]_n_-OH, (c) [Ni_2_(tmd)_2_]_n_-OH after 150-cycle CV scan, and (d) NiPc.

***Figure S11***. Ni K-edge EXAFS spectra (dots) and fitting (lines) of [Ni_2_(tmd)_2_]_n_, [Ni_2_(tmd)_2_]_n_-OH, and the samples after CV scans for different cycles.

***Table S5.*** Structural parameters from Ni K-edge EXAFS fitting for different CV cycles.

|  | path | R (Å) | N | △E (eV) | σ^2^(Å ^2^) | R factor |
| --- | --- | --- | --- | --- | --- | --- |
| [Ni_2_(tmd)_2_]_n_-OH | Ni-O | 1.744 | 2.004 | −0.039 | 0.004 | 0.001 |
|  | Ni-N(S) | 1.946 | 2.053 | -9.829 | 0.003 |  |
|  | Ni-N(L) | 2.302 | 1.090 |  |  |  |
| 1^st^ CV | Ni-O | 1.744 | 2.004 | -15.530 | 0.003 | 0.002 |
|  | Ni-N(S) | 1.946 | 2.053 | -9.829 | 0.002 |  |
|  | Ni-N(L) | 2.303 | 1.090 |  |  |  |
| 10^th^ CV | Ni-O | 1.753 | 2.004 | -15.540 | 0.002 | 0.002 |
|  | Ni-N(S) | 1.943 | 2.051 | -9.814 | 0.003 |  |
|  | Ni-N(L) | 2.300 | 1.090 |  |  |  |
| 30th CV | Ni-O | 1.743 | 1.994 | -15.525 | 0.002 | 0.001 |
|  | Ni-N(S) | 1.943 | 2.035 | -9.604 | 0.003 |  |
|  | Ni-N(L) | 2.299 | 1.090 |  |  |  |
| 50^th^ CV | Ni-O | 1.743 | 1.992 | −15.525 | 0.002 | 0.001 |
|  | Ni-N(S) | 1.945 | 2.041 | -9.580 | 0.003 |  |
|  | Ni-N(L) | 2.300 | 1.081 |  |  |  |
| 100th CV | Ni-O | 1.743 | 1.992 | −15.423 | 0.002 | 0.013 |
|  | Ni-N(S) | 1.945 | 2.041 | -9.580 | 0.003 |  |
|  | Ni-N(L) | 2.298 | 1.081 |  |  |  |
| 150^th^ CV | Ni-O | 1.796 | 2.030 | -7.191 | 0.003 | 0.001 |
|  | Ni-N(S) | 1.954 | 2.032 | -9.037 | 0.002 |  |
|  | Ni-N(L) | 2.237 | 1.074 |  |  |  |

Fitting was done across the *k* range of 2.25 to 9 Å^–1^ and the *R* range of 1.1 to 2.8 Å for all samples. Where N is the coordination number, R is the distance between the absorber and backscatter atoms, σ^2^ is the Debye-Waller factor, and R_f_ is the R-factor characterizing the goodness of fitting. Error bounds (accuracies) characterizing the structural parameters obtained by EXAFS data analysis are estimated to be as follows: N, ±20%; R, ±1%; and σ^2^, ±20%.

***Figure S12.*** N 1s spectra of [Ni_2_(tmd)_2_]_n_ and [Ni_2_(tmd)_2_]_n_-CV.

***Table S6.*** Ni-N_Py/Im_, N-N, Ni–N_amine_, and NO_x_ percent area distributions and their ratios in N 1s.

| N 1s Area Distribution (%) | Ni–N_Py/Im_ | N–N | | Ni–N_amine_ | | NO_x_ |
| --- | --- | --- | --- | --- | --- | --- |
| [Ni_2_(tmd)_2_]_n_ | 33.72 | 16.72 | | 33.14 | | 16.42 |
| [Ni_2_(tmd)_2_]_n_-CV | 38.7 | 11.63 | | 27.09 | | 22.58 |
| N-group Area Ratio | N–N/ Ni–N_Py/Im_ | | Ni–N_amine_/ Ni–N_Py/Im_ | | NO_x_/ Ni–N_Py/Im_ | |
| [Ni_2_(tmd)_2_]_n_ | 0.496 | | 0.983 | | 0.487 | |
| [Ni_2_(tmd)_2_]_n_-CV | 0.301 | | 0.700 | | 0.583 | |


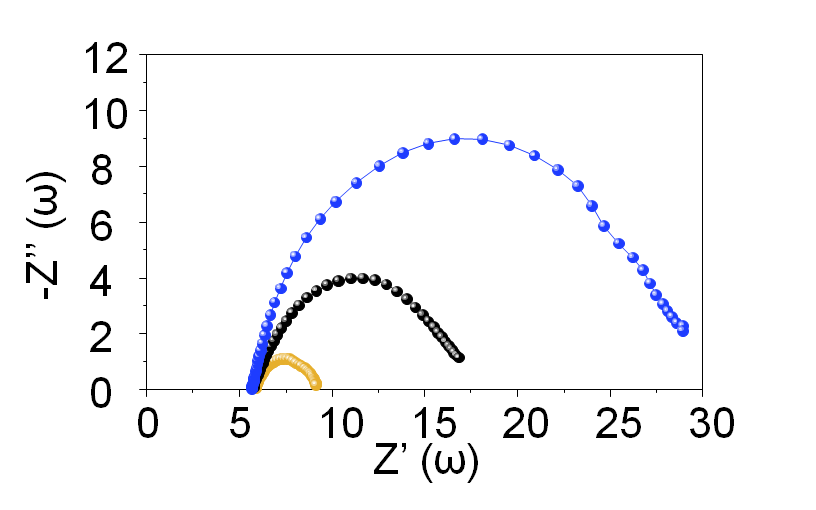


***Figure S13.*** Nyquist plot for NiPc (blue), RuO_2_ (black), and [Ni_2_(tmd)_2_]_n_-CV (orange) at 1.65 V vs RHE.

***Table S7.*** EIS fitting results of different electrocatalysts for OER at 1.65 V (V vs RHE).

|  | **R_s_ (Ω)** | **R_ct_ (Ω)** | **CPE_T1_**  **(mFs^α-1^)** | **CPE_P1_**  **(= α)** |
| --- | --- | --- | --- | --- |
| [Ni_2_(tmd)_2_]_n_-CV | 5.9 | 3.1 | 0.0025 | 0.79 |
| NiPc | 5.7 | 31.1 | 0.009 | 0.81 |
| RuO_2_ | 5.6 | 11.2 | 0.003 | 0.77 |
| NiO | 6.1 | 85.8 | 0.021 | 0.91 |

***Figure S14.*** Bode plot for [Ni_2_(tmd)_2_]_n_-CV by EIS measurements.

***Figure S15.*** CVs at different scan rates for (a) [Ni_2_(tmd)_2_]_n_-CV, (b) NiPc, (c) RuO_2_, and (d) NiO.

***Figure S16.*** Durability tests on [Ni_2_(tmd)_2_]_n_-CV, NiPc, RuO₂, and NiO with an initial current density of 50 mA/cm^2^. The remaining current density after 24 hours are 86%, 9%, 39%, and 8.6%, respectively.

***Figure S17.*** (a) The TEM image of [Ni_2_(tmd)_2_]_n_-CV after durability test at 1.63 V for 24 hours, and its corresponding EDS maps of (b) N, (c) O, and (d) Ni.

***Table S8.*** STEM-EDS semi-quantitative analysis of [Ni_2_(tmd)_2_]_n_ and [Ni_2_(tmd)_2_]_n_-CV after stability.

|  | N atom (%) | O atom (%) | Ni atom (%) | N/Ni | O/Ni |
| --- | --- | --- | --- | --- | --- |
| [Ni_2_(tmd)_2_]_n_ | 53.12 | 38.51 | 8.37 | 6.35 | 4.60 |
| [Ni_2_(tmd)_2_]_n_-CV  After stability | 49.67 | 44.53 | 5.80 | 8.56 | 7.68 |

***Figure S18.*** Post-durability XRD pattern of [Ni_2_(tmd)_2_]_n_ after 50 hours of OER electrolysis.

***Figure S19.*** Ni K-edge XANES spectra of (a) [Ni_2_(tmd)_2_]_n_-CV and after stability. (b) The enlarged region is the near-edge. After the stability test, it shifts toward higher photon energy by about 0.4 eV.

***Figure S20.*** Ni K-edge (a) EXAFS spectra for [Ni_2_(tmd)_2_]_n_-CV and after stability. (b) EXAFS fitting results for after stability.

***Figure S21.*** FDMNES simulations of [Ni_2_(tmd)_2_]_n_-OER.

***Figure S22.*** Ni K-edge EXAFS fitting results for (a) 1.45 V, (b) 1.53 V, (c) 1.60 V.

***Table S9.*** Structural parameters from Ni K-edge EXAFS fitting for different potentials.

|  | path | R (Å) | N | △E (eV) | σ^2^(Å ^2^) | R factor |
| --- | --- | --- | --- | --- | --- | --- |
| 1.45 V | Ni-O | 1.817 | 1.933 | −17.571 | 0.006 | 0.035 |
|  | Ni-O_ads_ | 2.611 | 0.562 |  |  |  |
|  | Ni-N(S) | 1.824 | 1.954 | -12.111 | 0.003 |  |
|  | Ni-N(L) | 2.345 | 1.093 |  |  |  |
| 1.53 V | Ni-O | 1.825 | 1.895 | -9.364 | 0.006 | 0.037 |
|  | Ni-O_ads_ | 2.627 | 0.617 |  |  |  |
|  | Ni-N(S) | 1.861 | 2.003 | -16.462 | 0.003 |  |
|  | Ni-N(L) | 2.281 | 1.053 |  |  |  |
| 1.6 V | Ni-O | 1.832 | 1.892 | -9.920 | 0.006 | 0.034 |
|  | Ni-O_ads_ | 2.674 | 0.713 |  |  |  |
|  | Ni-N(S) | 1.856 | 1.904 | -15.742 | 0.003 |  |
|  | Ni-N(L) | 2.296 | 1.055 |  |  |  |
| After  Durability Test | Ni-O | 1.797 | 2.138 | -7.191 | 0.002 | 0.001 |
|  | Ni-N(S) | 1.951 | 1.894 | -9.611 | 0.002 |  |
|  | Ni-N(L) | 2.227 | 0.923 |  |  |  |

Fitting was done across the *k* range of 2.25 to 9 Å^–1^ and the *R* range of 1.1 to 2.8 Å for 1.45 V, 1.53 V and 1.6V.  *k* range of 2.25 to 9 Å^–1^ and the *R* range of 1.1 to 2.8 Å for after stability.

***Figure S23.*** *In-situ* XES spectra of (a) [Ni_2_(tmd)_2_]_n_, [Ni_2_(tmd)_2_]_n_-CV, and under 1.45 V vs RHE. (b) Ligand field splitting patterns for 6 and 4-coordinated Ni^2+^ complexes.
